# Polyethylene eye-cover versus artificial teardrops in the prevention of ocular surface diseases in comatose patients: A prospective multicenter randomized triple-blinded three-arm clinical trial

**DOI:** 10.1371/journal.pone.0248830

**Published:** 2021-04-01

**Authors:** Mahnaz Khatiban, Hamid Moradi Amin, Gholamhosein Falahinia, Abbas Moghimbeigi, Mehran Yadollahi

**Affiliations:** 1 Department of Medical-Surgical Nursing, Department of Ethics Education in Medical Sciences, Mother & Child Care Research Center, School of Nursing and Midwifery, Hamadan University of Medical Sciences, Hamadan, Iran; 2 Students Research Center, School of Nursing and Midwifery, Hamadan University of Medical Sciences, Hamadan, Iran; 3 Department of Medical-Surgical Nursing, Chronic Disease (Home Care) Research Center, School of Nursing and Midwifery, Hamadan University of Medical Sciences, Hamadan, Iran; 4 Department of Biostatistics and Epidemiology, Modeling of Noncommunicable Disease Research Canter, School of Public Health, Hamadan University of Medical Sciences, Hamadan, Iran; 5 Malayer Mehr Hospital, Hamadan University of Medical Sciences, Hamadan, Iran; University of California, UNITED STATES

## Abstract

**Background:**

Polyethylene covers are claimed to be useful in preventing ocular surface diseases (OSD); however, evidence of their clinical efficacy is limited. This clinical trial aimed to compare the use of polyethylene eye covers and artificial teardrops versus normal saline on the incidence and severity of OSD in comatose patients.

**Methods:**

Of 90 eligible patients randomly assigned to three treatment groups, 79 patients completed the study, In group A, patients (n = 25) received artificial teardrops for left and normal saline for right eyes, in group B (n = 29) polyethylene covers for left and normal saline for right eyes, and in group C (n = 25) polyethylene covers for left and artificial teardrops for right eyes. As the patients were comatose, their blinding did not applicable, and a blinded observer evaluated the patients’ eyes based on the Corneal Fluorescein Staining Pattern. The blinded analyzer analyzed collected data by SPSS-16 software at a 95% confidential level.

**Results:**

The OSDs were observed in 65 (41.14%) out of 158 eyes examined. The artificial teardrop was more effective than the normal saline in group A, polyethylene eye cover was more useful than the normal saline in group B, and polyethylene eye cover was more effective than the artificial teardrop in group C in reducing the incidence of OSD (p< 0.01). Polyethylene eye covers had the most impact on reducing the severity of the OSD compared to the other interventions (p< .001).

**Conclusions:**

Polyethylene eye covers significantly reduced the incidence and severity of OSD. Using polyethylene cover is suggested as a safe, effective, and accessible eye care intervention for preventing OSD in comatose patients.

**Trial registration:**

(IRCT201609129014N115), Iranian Registry of Clinical Trials.

## Introduction

Patients who are admitted to Intensive Care Units (ICUs) usually experience failures in one or more vital organ systems such as respiratory, neurologic, and cardiovascular systems accompanied by many other complications. Ocular Surface Disease (OSD) is a common complication in this patient population, but receive minimal clinical consideration to prevent it [1, 2]. Due to the ICU patients’ life-threatening conditions, the eye care and symptoms of the OSD are often given low priority. OSD indicates damage to the surface layers of the eye, specifically, the corneal epithelial and conjunctiva, which is clinically diagnosed based on the slit lamp examination using different dyes such as fluorescein [3]. OSD is associated with different risk factors, with the most common being impaired secretion and distribution of tear film over the ocular surface, altered tear components concentration due to lack of blinking, incomplete lid closure (lagophthalmos), and lid or conjunctival edema occurring with the use of ventilator [4–9]. There is a range of eye complications from mild Conjunctival infections to severe ocular damage. Keratopathy (3.6% to 60%), chemosis (9% to 80%), and microbial keratitis are the most prevalent ocular disorders in ICU patients [10]. Insufficient eye care of ICU patients can increase the risk of serious eye problems such as corneal abrasions and ulceration, infectious keratitis, and even corneal perforation and loss of vision [11, 12].

It is highly recommended to integrate eye care into the treatment plan for critically ill patients in order to protect their eyes from OSDs. In recent decades, many evidence-based practices for eye care were developed; these practices include a wide range of interventions such as 1) washing the eyes with normal saline solution; 2) using lubricating ointments or teardrops; 3) moisturizing the chambers by applying a polyethylene eye cover, swimming goggles; 4) covering the eyes with eye shields, pads, or patches; and 5) closing the eyelid with closure by transparent tape or tarsorrhaphy. There is no clear evidence as to which of these interventions represent the ideal eye care for ICU patients to prevent OSD [4, 5, 9, 11, 13–18]. Accordingly, healthcare professionals’ beliefs and traditions have played a major role in selecting eye care interventions in the ICU settings in different countries [5, 17]. Applying polyethylene eye covers has been suggested as the most effective intervention [5] and the main components of the evidence-based protocol to prevent exposure keratopathy in the ICU settings [19]. But, in a very recent study of eye care for the intensive care unit, the lubricants and taping of the lids are only advised for the conjunctival and corneal exposure [18]. The polyethylene eye cover was not considered as a part of the standard eye care for critically ill patients as well. Inconsiderately, only has been reported a daily washing of eyelids with normal saline and sterile gauze and application of ocular lubricants at least twice per day as the standard care in ICUs [18].

In Iran, washing out eye discharge with sterile normal saline is considered as part of routine eye care for ICU patients, but this intervention is not well supported empirically and is no longer recommended worldwide. Our research team collaborated with health professionals to select alternative eye care interventions to prevent OSD in ICU patients. The team members carefully reviewed empirical and clinical literature to determine the effectiveness or benefits and risks of alternative interventions, while also considering their feasibility in the local ICU context. The results of this critical appraisal indicated the following: 1) Irrigating eye with sterile normal saline could cause an increase in the incidence of OSD and cross-infection, and the cotton wool balls can cause corneal scratching [5]. 2) lubrication materials could be hard and time-consuming to apply on regular or frequent time intervals by nurses [20]; further, the ointment containers can be infected if not used properly. 3) Using eyelid sutures makes regular eye checkups difficult; the sutures are also unpleasant in appearance and block the eye activities [20]. 4) Tarsorrhaphy is also invasive and almost impossible to apply to our lagophthalmos patients. 5) Moisture chamber goggles are only designed for adults and are inconvenient to use when patients are in a supine position [20]. Securing the moisture chamber goggles around the head also increases the risk of edematous eyes in ventilated patients. 6) Mechanical closure of the eyes is recommended in the case of lagophthalmos [21]. 7) Polyethylene covers may be pulled by infants, children, or agitated patients [20]. Lastly, eye covering is claimed as the most effective intervention in preventing exposure keratopathy [5].

After examining the benefits and risks of each eye care interventions and its feasibility in our ICU settings, the research team decided to use polyethylene eye covers. However, evidence to support their clinical efficacy in reducing the incidence and severity of OSD is limited.

### Research objectives

This clinical trial was carried out to compare the use of polyethylene eye covers, artificial teardrops, and normal saline drops on the incidence and severity of OSD in comatose patients admitted to the ICU.

## Materials and methods

### Setting

The study was carried out in the three ICUs in university-affiliated hospitals located in Malayer, Iran over an eight-month period, from September 2016 to May 2017. The units had comparable bed capacity, providing care to patients with a range of surgical and medical conditions, including coma.

### Ethical approval

This study was carried out with the ethical standards set forth in the Helsinki Declaration of 1975, reviewed and approved by the Ethical Committee affiliated to the Research and Technology Vice-chancellor of Hamadan University of Medical Sciences (IR.UMSHA.REC.1395.262), and was registered on the Iranian Registry of Clinical Trials (IRCT No: IRCT201609129014N115). The written consents were signed by the patient’s family after explaining the aims and objectives of the study and their right to declining the study without any negative consequences.

### Study design

This was a prospective multicenter randomized triple-blinded three-arm clinical trial. After considering three groups where every patient may be allocated, to control for possible between-participant differences in clinical conditions that may influence the eyes’ status, randomization was done at two levels. First, eye care interventions were randomly allocated to one of the left or right eye for each participant in each group, by tossing a coin as detailed next.

- Group “A” patients received artificial teardrops for the left eyes and normal saline for the right eyes.
- Group “B” patients received polyethylene cover for the left eyes and normal saline for the right eyes.
- Group “C” patients received polyethylene cover to the left eyes and artificial teardrops to the right eyes.

Second, eligible participants were assigned to one of three treatment groups by permuted block randomization (AABBCC, ABCABC, BBCCAA …) enveloped in the opaque packets and selected by a nurse outside the study. The sample consisted of patients newly admitted to the participating ICUs. Patients were enlisted if only they were over 18 years of age, comatose (with a Glasgow Coma Scale (GCS) less than or equal to 8 of 15), mechanical ventilated and who had no trauma to the areas of the face and eyes and no history of chronic disease, electrolyte imbalance, cataract surgery or glaucoma. An ophthalmologist confirmed that the patients had a lack/ difficulty in blinking (less than five times per minute), incomplete eyelid closure, and a healthy cornea after examination by the fluorescein and a portable slit lamp. Patients who showed signs of consciousness or natural blinking reflexes required cardiopulmonary resuscitation during the trial and got discharged from ICUs to the other centers or died, were excluded.

As the patients were comatose, their blinding did not apply. A single observer who was blinded to the patient assignments evaluated the patients’ eyes based on the Corneal Fluorescein Staining Pattern. The data analyst blinded to the allocated treatment analyzed the primary outcome.

The sample size was calculated according to the formula below. With an 80% power and a 2-sided significance level of α = .05, the number of people needed in each group was 25. Anticipating a total attrition rate of 20%, a total of 90 patients were selected and included in the study, 30 patients for each arm.

$$n={\left[\frac{\left(Z_{1-\alpha /2}\sqrt{p\left(1-p\right)}\right)+\left(Z_{1-\beta }\sqrt{p_{2}\left(1-p_{2}\right)}\right)}{d}\right]}^{2}$$

Of the 90 eligible patients, 79 patients completed the trial participation. The number of patients in group A was 25, group B came to 29, and group C remained 25. In a post hoc power analysis by G*Power 3.1.9.2 software, when we set a small effect size of 0.25, a significance level of α = .05, with a total sample size of 158 in three groups, the achieved power computed more than 0.80.

### Interventions

All patients received routine eye care. In all participating ICUs, routine eye care consisted of eye irrigation with sterile normal saline when necessary (PRN). In addition to usual care, participants in the three treatment groups received the intervention allocated to each eye. Prior to applying the intervention, the eyelids and surrounding skin were gently irrigated by sterile normal saline and sterile gauze in the same way.

For the intervention with artificial teardrops, while pulling down the lower lid of the patient’s eye to form a V pocket, two Tearlose (Miscellaneous) drops were applied every six hours. Tearlose is a sterile eye drop with the generic name of Polyvinyl Alcohol or Hydroxypropyl Methylcellulose which contained Cellulose 0.3g/100mL and Dextran 70g/100mL. For the intervention with normal saline, two drops of saline 0.9% were applied in the same manner and dose. The sterile ophthalmic solution of sodium chloride is in a 5-milliliter unit-dose made in a validated company in Iran. For the intervention with a polyethylene eye cover, 2.5-inch square pieces of thin plastic film were put from the above of the eyebrow to the cheekbone and fixed by hypoallergenic paper tape [22] every 12 hours. To ensure corneal oxygenation, the patients’ eyes were left uncovered for 15 minutes before the next eye covering. During this time, the patient’s eyelid was slightly opened and closed several times. All interventions were provided over a 5-day period because the ocular surface changes are established in the early days of hospitalization and the average time of about five days (4.55±2.97) is enough for the development of the OSD in ICU patients [23].

### Data collection

Data were collected on patients’ characteristics at baseline, and on the outcomes of incidence and severity of OSD at baseline and post-intervention.

1. Patients’ demographic and clinical characteristics. These included age, sex, and reason for ICU admission. Pertinent data were extracted from their medical records.

2-Outcomes. These were assessed with the Corneal Fluorescein Staining Pattern. The corneal fluorescein staining is a valuable clinical tool to examine the viability of the epithelium [24] and is able to provide extensive knowledge about the ocular surface [25]. As this clinical ocular examination is introduced as a feasible, cheap, safe, acceptable, reasonably sensitive, and specific in identifying corneal damage in ICU patients as well [26], fluorescein testing was considered to measure and classify the patients’ OSD. At the portable slit-lamp microscope with a blue filter, the cornea was examined between four and eight minutes following the instillation of Fluorescein Sodium 1% eye drops. To spread the Fluorescein over the entire surface of the eye, the patient’s eyelid was gently opened and closed a few times to spread the Fluorescein on the eye surface. The concentration and the breadth of the corneal staining provide valuable evidence to measure disease severity and to monitor the response to treatment [24]. Punctate epithelial erosions (PEE) are counted and scored from zero to six, the maximum possible score for each cornea. Grade 0 for no PEE, grade 1 for 1–5 PEE involving the inferior third of the cornea, grade 2 for 6–30 PEE involving more than the inferior third of the corneal surface, and grade 3 for more than 30 PEE, macroepithelial defect. A score of severity is added when the PEE is seen in the central 4mm diameter portion of the cornea presenting the stromal whitening of the epithelial defect, grade 4. When one or more filaments occurred anywhere on the cornea, stromal scar, the grade is 5. Grade 6 is considered if one or more patches of confluent staining including linear stains, microbial keratitis, found anywhere on the cornea [20, 27]. An ophthalmologist, unaware of patients’ groups, graded their corneas according to the Corneal Fluorescein Staining Pattern at the baseline and after two hours of the last intervention on the fifth day. In order to mask the examiner, the eye covers were removed before the last eye examination.

### Statistical analysis

In addition to descriptive statistics, the assumption of normality for numerical variables was examined with the Kolmogorov-Smirnov test. Patients’ characteristics were compared across the three treatment groups, using the Chi-Square test for categorical variables (sex, and type of injury) and one-way Analysis of variance (ANOVA) for numerical variables (age). The incidence of OSD was compared across the three treatment groups by McNemar’s test and the severity of the OSD by the Kruskal–Wallis test. The Statistical Package for the Social Sciences version 16.0 for Windows (SPSS Inc., Chicago, Illinois) was used for the data analysis at a 95% confidence level for all tests.

## Results

**Fig 1:**
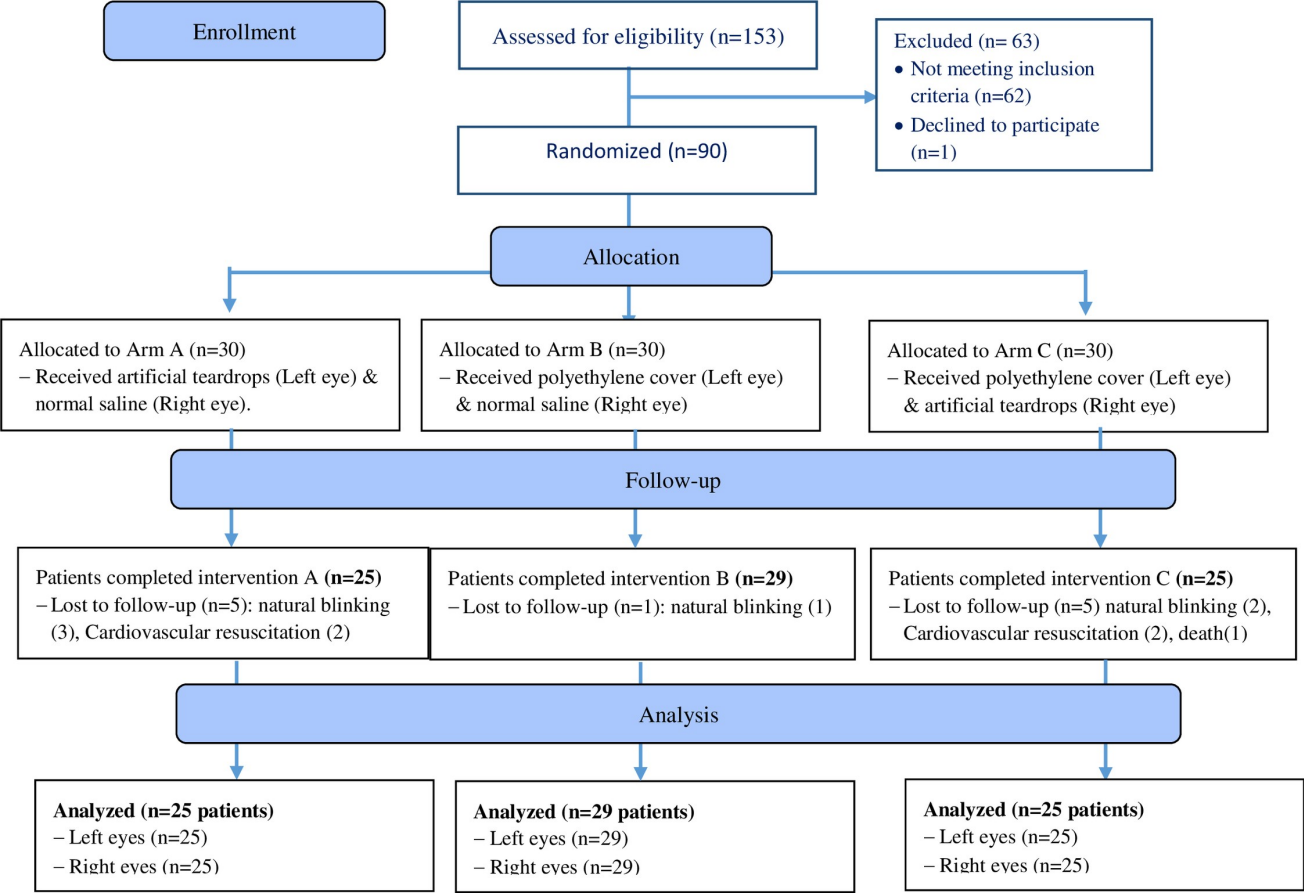
CONSORT flow diagram.

In this study, the data of 158 eyes were compared, 54 eyes for normal saline, 50 for artificial teardrops, and 54 for polyethylene cover interventions (Fig 1).

Patients assigned to the three treatment groups were comparable on demographic and clinical characteristics (all p’s > .05) (Table 1). The majority of patients were older men, admitted for the management of Neurology diseases.

**Table 1:** Comparison of the studied demographic characteristics among groups (n = 90).

Variable		Group A (n = 30)	Group B (n = 30)	Group C (n = 30)	p-value
Gender, n (%)	Male	20 (66.7%)	19 (63.3%)	17 (56.8%)	.718 ^A^
	Female	10 (33.3%)	11 (36.7%)	13 (43.3%)	
Reason of ICU admission, n (%)	Neurologic patients	18 (60.0%)	24 (80.0%)	21 (70.0%)	.207 ^B^
	Internal medicine patients	10 (33.3%)	6 (20.0%)	9 (30.0%)	
	Surgical patients	2 (0.60%)	0 (0.0%)	0 (0.0%)	
Age (M±SD)		63.17±20.86	63.20±16.52	68.20±16.62	.468 ^C^

^A^ Chi-square test.

^B^ Fisher’s exact test.

^C^ One-way ANOVA.

### Incidence of ocular surface disease

The presence of OSD in each eye, in patients assigned to the three treatment groups, is shown in Table 2. The OSDs were observed in 65 (41.14%) out of 158 eyes examined. McNemar’s test compared the presence of OSD across groups. The results indicated that in group A, the artificial teardrops intervention was considerably more useful than the sterile normal saline drops intervention (p< .01). In group B, the polyethylene cover intervention was significantly better than the sterile normal saline drops intervention (p< .001). Finally, in group C, the polyethylene cover intervention was also better than the artificial teardrops intervention (p< .01).

**Table 2:** Comparison of the patients’ eyes according to the incidence of the ocular surface disease.

Group		Incidence of the ocular surface disease		^D^ p-value
		Yes	No	
**Group A (n = 25)**	Right eye (Normal saline drops)	18 (72.0%)	7 (28.0%)	.002
	Left eye (Artificial teardrops)	9 (36.0%)	16 (64.0%)	
**Group B (n = 29)**	Right eye (Normal saline drops)	19 (65.5%)	10 (34.5%)	.001
	Left eye (Polyethylene cover)	5 (17.2%)	24 (82.8%)	
**Group C (n = 25)**	Right eye (Artificial teardrops)	11 (44.0%)	14 (56.0%)	.008
	Left eye (Polyethylene cover)	3 (12.0%)	22 (88.0%)	
	**Total**	65 (41.14%)	93 (58.86%)	

^D^ Results of McNemar’s test.

### Severity of ocular surface disease

The severity grading of the OSDs according to the Corneal Fluorescein Staining Pattern in three treatment groups is shown in Table 3. Although the artificial teardrops intervention was better than normal saline drops intervention, Kruskal–Wallis test showed that the polyethylene eye covers intervention was the best in reducing the severity of OSD (p < .001).

**Table 3:** Comparison of the patients’ eyes according to the severity of the ocular surface disease.

Eye treatment	Grading*							(M±SD)	^E^ p-value
	0	1	2	3	4	5	6		
**Normal saline drops (n = 54 eyes)**	17 (31.5%)	5 (9.3%)	17 (31.5%)	10 (18.5%)	4 (7.4%)	1 (1.5%)	0 (0.0%)	1.60±1.38	< .001
**Artificial teardrops (n = 50 eyes)**	28 (56.0%)	12 (20.0%)	10 (16.7%)	0 (0.0%)	0 (0.0%)	0 (0.0%)	0 (0.0%)	0.64±0.80	
**Polyethylene covers (n = 54 eyes)**	47 (87.0%)	6 (11.1%)	1 (1.9%)	0 (0.0%)	0 (0.0%)	0 (0.0%)	0 (0.0%)	0.15±0.41	

* Grading: Grade 0 for no punctate epithelial erosions (PEE), Grade 1 for 1–5 PEE, Grade 2 for 6–30 PEE, and Grade 3 for more than 30 PEE. A score of severity is added when the PEE was seen in the central 4mm diameter portion of the cornea, one or more filaments occurred anywhere on the cornea, or one or more patches of confluent staining, including linear stains, are found anywhere on the cornea.

^E^ Results of Kruskal–Wallis test.

## Discussion

In this clinical trial, three eye care interventions, applied over a five-day period were compared. The findings showed that the artificial teardrops intervention is more effective than the normal saline drops intervention, and the polyethylene eye cover is the best intervention for reducing the incidence and severity of OSD in comatose patients. Moreover, eye covering with a transparent thin plastic film was not a barrier for frequent observation and monitoring of the cornea. Our results reconfirmed the effectiveness of the polyethylene eye-cover. Granting that polyethylene covering is recommended as an effective eye-care in ICU patients [28], the method of using it is not explained in detail. This is the first study for the ICU patient’s eye care in which the eye covering was precisely defined, every 12 hours with considering a 15-minute of the cornea oxygenation in each eye re-covering. So, our procedure of polyethylene eye covering is able to improve the evidence-based eye-care in ICU patients.

Our findings are also consistent with those reported by Shan and Min in showing that polyethylene eye covers maintain a healthy cornea; the polyethylene cover fared better than the moist chamber or artificial teardrops [29]. Using polyethylene eye covers every 12 hours has also been compared to Carbomer eye drops applied every six hours for a period of five days; the polyethylene cover was found to significantly prevent the dry eye syndrome in unconscious patients [30]. Also, polyethylene covers were significantly more beneficial in the prevention of keratopathy than Liposic ophthalmic ointment and artificial teardrops [31]. Polyethylene eye covers are more effective than the hypromellose and Lacri-Lube combination in reducing the incidence of corneal ulceration in intensive care patients [32].

The effectiveness of polyethylene covers is not only related to the simple eye closing mechanism. The results of a study showed that using wet gauze to cover the eye was not significantly useful in preventing dry eye and corneal ulcers in patients during general anesthesia [7]. Taping eyes is another method of eye closing that was reported to be more effective than the eye gel membrane in preventing the development of keratopathy in comatose patients. Taping the eye and eyelid has been advocated as a safe prophylactic method, even for patients who require frequent pupillary examination [33]; however, the risk of injury to the eyelids and nearby skin and the probability of increasing the relatives’ distress remain [34].

Our study results support the usefulness of covering eyes with a polyethylene film in the prevention of OSD; polyethylene eye covers simultaneously protect eyes by creating a moist chamber, provide a naturally lubricated eye surface by preventing the evaporation of tears, and stop the growth of microorganisms in patient’s eyes, especially during airway suctioning and tube feeding procedures. In a comparison of the two eye care interventions, polyacrylamide hydrogel dressings (Geliperm) versus an ocular lubricant (Lacrilube), Geliperm was found as effective as Lacrilube in preventing exposure keratopathy in ICU patients [35]. The polyacrylamide hydrogel dressings provide a moist environment at the surface of the wound, are resistant to bacteria, but permeable to water vapor, gases, and small protein molecules. Although these dressings are a means for eye closing, their permeability makes them an ocular lubricant in the eye care of comatose patients.

Using polyethylene eye cover can minimize the frequency of eye cleansing by swab wash. Eye irrigation with sterile water/normal saline-soaked cotton balls or gauze is typically recommended as a hygiene regimen or regular cleansing of the eye for critical patients in ICUs [18, 36], but eye irrigation increases the incidence of OSD and infection, and corneal scratching [5]. Covering eyes with polyethylene every 12 hours can save time on care and decrease threats of eye abrasion and infection. In our study, the eyes covered with polyethylene have usually remained clean after 12 hours of the previous cover exchange.

The finding of this clinical trial and of another study indicate that polyethylene covers are effective, time-saving, and easy intervention for eye care in critically ill patients [29]. So, it is seriously recommended to the ICU nurses to substitute applying normal saline drops/irrigation with polyethylene eye covering. The limitation of this study relates to the evaluation of symptoms of OSD in comatose patients. Further research is needed to examine the eye care intervention in conscious patients who are not able to close their eyelids well, such as Bell’s palsy, severe exophthalmos, and severe skin disorders as ichthyosis. Research with this patient population would provide a good opportunity to inquire about patients’ perspectives on different eye care interventions. In this study, we measured the incidence and severity of the punctate epithelial erosions of the cornea. So, the other forms of OSDs in ICU patients such as epithelial defects, infections, impaired secretion and distribution of tear film over the ocular surface, tear components concentration, and conjunctival manifestations due to the use of the polyethylene eye covering and/or artificial teardrops need more studies. Although our eligible patients had incomplete eyelids closure and each patient was a member of the control and/or intervention group simultaneously, the grades of eye closure were not our main criterion. So, more studies are needed about the only cornea exposed eyes for more specific results. Future studies could also evaluate the initiative to implement pre-disposable polyethylene eye covers into nursing practice. A cost-effectiveness analysis may also be of relevance, given the decreased financial costs and less timewasting with the eye covering.

## Conclusion

In conclusion, Polyethylene eye cover demonstrated superior efficacy in preventing both incidence and severity of the OSD in comatose patients compared to normal saline and artificial teardrops. Adding a polyethylene eye cover to the routine care in critical care settings is necessary to prevent the OSD and subsequent complications.

## Supporting information

S1 Checklist CONSORT 2010 checklist of information to include when reporting a randomised trial*. (DOC)

S1 Table Comparison of the patients’ gender differences among three studied groups (n = 90). (DOCX)

S2 Table Comparison of the patients’ reasons of ICU admission among three studied groups (n = 90). (DOCX)

S3 Table Comparison of the patients’ age among three studied groups (n = 90). (DOCX)

S4 Table Comparison of the patients’ eyes according to the incidence of the ocular surface disease. (DOCX)

S5 Table Comparison of the Ocular Surface Disease (OSD) of the patients’ right eyes among three groups (total number of patients = 79). (DOCX)

S6 Table Comparison of the Ocular Surface Disease (OSD) of the patients’ left eyes among three groups (total number of patients = 79). (DOCX)

S7 Table Comparison of the Ocular Surface Disease (OSD) of the patients’ eyes among three groups. (DOCX)

S8 Table Comparison of the severity of the Ocular Surface Disease (OSD) in the patients’ right eyes among three groups (n = 79). (DOCX)

S9 Table Comparison of the severity of the Ocular Surface Disease (OSD) in the patients’ left eyes among three groups (n = 79). (DOCX)

S10 Table Comparison of the severity of the Ocular Surface Disease (OSD) of the patients’ right eyes (number of patients’ eyes = 79). (DOCX)

S11 Table Comparison of the severity of the Ocular Surface Disease (OSD) of the patients’ left eyes (number of patients’ eyes = 79). (DOCX)

S12 Table Comparison of the patients’ eyes according to the severity of the ocular surface disease (total number of patients’ eyes = 158). (DOCX)

S1 Protocol (DOCX)

S2 Protocol (DOCX)
