# Supplementary material for: Polyethylene eye-cover versus artificial teardrops in the prevention of ocular surface diseases in comatose patients: A prospective multicenter randomized triple-blinded three-arm clinical trial
Source: PLoS One. 2021 Apr 1;16(4):e0248830. doi: 10.1371/journal.pone.0248830 (PMC8016328; doi:10.1371/journal.pone.0248830)
Supplement: S1 Table — (DOCX) [file pone.0248830.s002.docx]

**S1 Table: Comparison of the patients’ gender differences among three studied groups (n = 90)**

| **Gender** | **Group A (n=30)** | **Group B (n=30)** | **Group C (n=30)** | **Chi-square test** |
| --- | --- | --- | --- | --- |
| **Male** | 20 (66.7 %) | 19 (63.3 %) | 17 (56.8 %) | X^2^ = .662  p = .718 |
| **Female** | 10 (33.3 %) | 11 (36.7 %) | 13 (43.3 %) |  |
