# Supplementary material for: Polyethylene eye-cover versus artificial teardrops in the prevention of ocular surface diseases in comatose patients: A prospective multicenter randomized triple-blinded three-arm clinical trial
Source: PLoS One. 2021 Apr 1;16(4):e0248830. doi: 10.1371/journal.pone.0248830 (PMC8016328; doi:10.1371/journal.pone.0248830)
Supplement: S2 Table — (DOCX) [file pone.0248830.s003.docx]

**S2 Table: Comparison of the patients’ reasons of ICU admission among three studied groups (n = 90)**

| **Reasons of ICU admission, n (%)** | **Group A (n=30)** | **Group B (n=30)** | **Group C (n=30)** | **Fisher’s exact test** |
| --- | --- | --- | --- | --- |
| **Neurologic** | 18 (60.0 %) | 24 (80.0 %) | 21 (70.0 %) | X^2^ = 5.897  p = .207 |
| **Internal patients** | 10 (33.3 %) | 6 (20.0 %) | 9 (30.0 %) |  |
| **Surgical patients** | 2 (0.60 %) | 0 (0.0 %) | 0 (0.0 %) |  |
