# Supplementary material for: Polyethylene eye-cover versus artificial teardrops in the prevention of ocular surface diseases in comatose patients: A prospective multicenter randomized triple-blinded three-arm clinical trial
Source: PLoS One. 2021 Apr 1;16(4):e0248830. doi: 10.1371/journal.pone.0248830 (PMC8016328; doi:10.1371/journal.pone.0248830)
Supplement: S3 Table — (DOCX) [file pone.0248830.s004.docx]

**S3 Table: Comparison of the patients’ age among three studied groups (n = 90)**

| **Variable** | **Group A (n=30)** | **Group B (n=30)** | **Group C (n=30)** | **One-way ANOVA** |
| --- | --- | --- | --- | --- |
| **Age** (M±SD) | 63.17±20.86 | 63.20±16.52 | 68.20±16.62 | F = .767  p = .468 |
