# Supplementary material for: Polyethylene eye-cover versus artificial teardrops in the prevention of ocular surface diseases in comatose patients: A prospective multicenter randomized triple-blinded three-arm clinical trial
Source: PLoS One. 2021 Apr 1;16(4):e0248830. doi: 10.1371/journal.pone.0248830 (PMC8016328; doi:10.1371/journal.pone.0248830)
Supplement: S4 Table — (DOCX) [file pone.0248830.s005.docx]

**S4 Table: Comparison of the patients’ eyes according to the incidence of the ocular surface disease**

| **Groups** |  | **Incidence of the ocular surface disease** | | **^D^ p-value** |
| --- | --- | --- | --- | --- |
|  |  | **Yes** | **No** |  |
| **Group A (n=25)** | Right eye (Normal saline drops) | 18 (72.0 %) | 7 (28.0 %) | .002 |
|  | Left eye (Artificial teardrops) | 9 (36.0 %) | 16 (64.0 %) |  |
| **Group B (n=29)** | Right eye (Normal saline drops) | 19 (65.5 %) | 10 (34.5 %) | .001 |
|  | Left eye (Polyethylene cover) | 5 (17.2 %) | 24 (82.8 %) |  |
| **Group C (n = 25)** | Right eye (Artificial teardrops) | 11 (44.0 %) | 14 (56.0 %) | .008 |
|  | Left eye (Polyethylene cover) | 3 (12.0 %) | 22 (88.0 %) |  |
|  | **Total** | 65 (41.14%) | 93 (58.86%) |  |

^D^ Results of McNemar's test
