# Supplementary material for: Polyethylene eye-cover versus artificial teardrops in the prevention of ocular surface diseases in comatose patients: A prospective multicenter randomized triple-blinded three-arm clinical trial
Source: PLoS One. 2021 Apr 1;16(4):e0248830. doi: 10.1371/journal.pone.0248830 (PMC8016328; doi:10.1371/journal.pone.0248830)
Supplement: S5 Table — (DOCX) [file pone.0248830.s006.docx]

**S5 Table: Comparison of the Ocular Surface Disease (OSD) of the patients’ right eyes among three groups (Total number of patients=79)**

| **Group** | **Right eye** | **Number of patients** | **OSD** | | **Chi-square test** |
| --- | --- | --- | --- | --- | --- |
|  |  |  | **Yes** | **No** |  |
| **A** | Normal saline drops | 25 | 18 (22.8 %) | 7 (8.9 %) | X^2^ = 4.45  p = .103 |
| **B** | Normal saline drops | 29 | 19 (24.1 %) | 10 (12.7 %) |  |
| **C** | Artificial teardrops | 25 | 11 (13.9 %) | 14 (17.7 %) |  |
