# Supplementary material for: Polyethylene eye-cover versus artificial teardrops in the prevention of ocular surface diseases in comatose patients: A prospective multicenter randomized triple-blinded three-arm clinical trial
Source: PLoS One. 2021 Apr 1;16(4):e0248830. doi: 10.1371/journal.pone.0248830 (PMC8016328; doi:10.1371/journal.pone.0248830)
Supplement: S6 Table — (DOCX) [file pone.0248830.s007.docx]

**S6 Table: Comparison of the Ocular Surface Disease (OSD) of the patients’ left eyes among three groups (Total number of patients=79)**

| **Group** | **Left eye** | **Number of patients** | **OSD** | | **Chi-square test** |
| --- | --- | --- | --- | --- | --- |
|  |  |  | **Yes** | **No** |  |
| **A** | Artificial teardrops | 25 | 9 (11.4 %) | 16 (20.3 %) | X^2^ = 6.32  p = .041 |
| **B** | Polyethylene cover | 29 | 5 (6.3 %) | 24 (30.4 %) |  |
| **C** | Polyethylene cover | 25 | 3 (2.67%) | 22 (29.1%) |  |
