# Supplementary material for: Polyethylene eye-cover versus artificial teardrops in the prevention of ocular surface diseases in comatose patients: A prospective multicenter randomized triple-blinded three-arm clinical trial
Source: PLoS One. 2021 Apr 1;16(4):e0248830. doi: 10.1371/journal.pone.0248830 (PMC8016328; doi:10.1371/journal.pone.0248830)
Supplement: S7 Table — (DOCX) [file pone.0248830.s008.docx]

**S7 Table: Comparison of the Ocular Surface Disease (OSD) of the patients’ eyes among three groups**

| **Eye treatment** | **Number of Eyes** | **OSD** | | **Chi-square test** |
| --- | --- | --- | --- | --- |
|  |  | **No** | **Yes** |  |
| **Normal saline drops** | 54 | 17 (31.5 %) | 37 (68.6 %) | < .001 |
| **Artificial teardrops** | 50 | 30 (60.0 %) | 20 (40.0 %) |  |
| **Polyethylene covers** | 54 | 46 (85.2 %) | 8 (14.8 %) |  |
