# Supplementary material for: Polyethylene eye-cover versus artificial teardrops in the prevention of ocular surface diseases in comatose patients: A prospective multicenter randomized triple-blinded three-arm clinical trial
Source: PLoS One. 2021 Apr 1;16(4):e0248830. doi: 10.1371/journal.pone.0248830 (PMC8016328; doi:10.1371/journal.pone.0248830)
Supplement: S8 Table — (DOCX) [file pone.0248830.s009.docx]

**S8 Table: Comparison of the severity of the Ocular Surface Disease (OSD) in the patients’ right eyes among three groups (n=79)**

| **Group** | **Right eye** | **Number of patients** | **Severity of OSD (0-6)** | | **Kruskal–Wallis test** |
| --- | --- | --- | --- | --- | --- |
|  |  |  | **Mean (±SD)** | **Mean Rank** |  |
| **A** | Normal saline drops | 25 | 1.52 (±1.23) | 49.92 | H = 8.94  p = .011 |
| **B** | Normal saline drops | 29 | 1.79 (±1.52) | 46.60 |  |
| **C** | Artificial teardrops | 25 | .72 (± .89) | 29.42 |  |
