# Supplementary material for: Polyethylene eye-cover versus artificial teardrops in the prevention of ocular surface diseases in comatose patients: A prospective multicenter randomized triple-blinded three-arm clinical trial
Source: PLoS One. 2021 Apr 1;16(4):e0248830. doi: 10.1371/journal.pone.0248830 (PMC8016328; doi:10.1371/journal.pone.0248830)
Supplement: S11 Table — (DOCX) [file pone.0248830.s012.docx]

**S11 Table: Comparison of the severity of the Ocular Surface Disease (OSD) of the patients’ left eyes (Number of patients’ eyes= 79)**

| **Left Eye treatment** | | **Grading*** n (%) | | | | | | | **Chi-square test** |
| --- | --- | --- | --- | --- | --- | --- | --- | --- | --- |
|  |  | **0** | **1** | **2** | **3** | **4** | **5** | **6** |  |
| **A** | Artificial teardrops (n=25) | 14  (56.0) | 8  (32.0) | 3  (12.0) | 0  (0.0) | 0  (0.0) | 0  (0.0) | 0  (0.0) | .011 |
| **B** | Polyethylene cover (n=29) | 24  (82.8) | 4  (13.8) | 1  (3.4) | 0  (0.0) | 0  (0.0) | 0  (0.0) | 0  (0.0) |  |
| **C** | Polyethylene cover (n=25) | 23  (92.0) | 2  (8.0) | 0  (0.0) | 0  (0.0) | 0  (0.0) | 0  (0.0) | 0  (0.0) |  |
| Total (n=79) | | 61 | 16 | 4 | 0 | 0 | 0 | 0 |  |

^*^ Grading: Grade 0 for no punctate epithelial erosions (PEE), Grade 1 for 1-5 PEE, Grade 2 for 6-30 PEE, and Grade 3 for more than 30 PEE. A score of severity is added when the PEE was seen in the central 4mm diameter portion of the cornea, one or more filaments occurred anywhere on the cornea, or one or more patches of confluent staining, including linear stains, are found anywhere on the cornea.
