# Supplementary material for: Polyethylene eye-cover versus artificial teardrops in the prevention of ocular surface diseases in comatose patients: A prospective multicenter randomized triple-blinded three-arm clinical trial
Source: PLoS One. 2021 Apr 1;16(4):e0248830. doi: 10.1371/journal.pone.0248830 (PMC8016328; doi:10.1371/journal.pone.0248830)
Supplement: S12 Table — (DOCX) [file pone.0248830.s013.docx]

**S12 Table: Comparison of the patients’ eyes according to the severity of the ocular surface disease (total number of patients’ eyes = 158)**

| **Eye treatment** | **Grading*** n (%) | | | | | | | **(M±SD)** | **Kruskal–Wallis test** |
| --- | --- | --- | --- | --- | --- | --- | --- | --- | --- |
|  | **0** | **1** | **2** | **3** | **4** | **5** | **6** |  |  |
| **Normal saline drops (n=54 eyes)** | 17  (31.5) | 5  (9.3) | 17  (31.5) | 10  (18.5) | 4  (7.4) | 1  (1.5) | 0  (0.0) | 1.60±1.38 | <.001 |
| **Artificial teardrops (n=50 eyes)** | 28  (56.0) | 12  (20.0) | 10  (16.7) | 0  (0.0) | 0  (0.0) | 0  (0.0) | 0  (0.0) | 0.64±0.80 |  |
| **Polyethylene covers (n=54 eyes)** | 47  (87.0) | 6 (11.1) | 1  (1.9) | 0  (0.0) | 0  (0.0) | 0 (0.0) | 0  (0.0) | 0.15±0.41 |  |

^*^ Grading: Grade 0 for no punctate epithelial erosions (PEE), Grade 1 for 1-5 PEE, Grade 2 for 6-30 PEE, and Grade 3 for more than 30 PEE. A score of severity is added when the PEE was seen in the central 4mm diameter portion of the cornea, one or more filaments occurred anywhere on the cornea, or one or more patches of confluent staining, including linear stains, are found anywhere on the cornea.
