# Supplementary material for: Polyethylene eye-cover versus artificial teardrops in the prevention of ocular surface diseases in comatose patients: A prospective multicenter randomized triple-blinded three-arm clinical trial
Source: PLoS One. 2021 Apr 1;16(4):e0248830. doi: 10.1371/journal.pone.0248830 (PMC8016328; doi:10.1371/journal.pone.0248830)
Supplement: S1 Protocol — (DOCX) [file pone.0248830.s014.docx]

**Effect of polyethylene film and artificial teardrops versus normal saline on the incidence of superficial ocular disorders in unconscious patients: a prospective randomized triple-blinded three-arm clinical trial**

**Background:** Polyethylene covers are claimed to be useful in preventing ocular surface diseases (OSD); however, evidence of their clinical efficacy is limited. This clinical trial aims to compare the effect of polyethylene film and artificial teardrops versus normal saline on the incidence of OSD in unconscious patients. This paper summarizes the study protocol.

**Method:** The design of the planned study is a multicenter randomized blinded three-arm clinical trial. Although we need 25 patients in each arm, anticipating a total attrition rate of 20%, a total of 90 patients will be selected and included in the study, 30 patients for each arm. We will set up three arms ‘A’, ‘B’, and ‘C’ in which each eye of a patient will be considered for one of three interventions: artificial teardrops, polyethylene covers, or normal saline eye drops by tossing a coin. Then, each eligible patient will be allocated to one of the three arms by permuted block randomization. All patients are unconscious so, their blinding does not apply. Both the eye observer and the analyzer will be unaware of the type of interventions. The patients’ eyes will be examined based on the Corneal Fluorescein Staining Pattern. The collected data will be analyzed by SPSS-16 software at a 95% confidential level.

**Discussion:** The results of this study will contribute to the developing pieces of evidence for a safe, effective, and accessible eye care intervention for preventing the incidence of OSD in comatose patients.

**Keywords**: Coma, Infectious Diseases, Eye, Intensive Care Units, Nursing Care, Patients.

1. **Introduction**

The Ocular Surface Disease (OSD) is a common complication in patients who are admitted to Intensive Care Units (ICUs) but to receive minimal clinical consideration to prevent it [1, 2]. OSD is associated with different risk factors, as the impaired secretion and distribution of tear film over the ocular surface, tear components concentration due to lack of blinking, incomplete lid closure (lagophthalmos), and lid or conjunctival edema occurring with the use of a ventilator [3-8]. For these patients, there is a range of eye complications from mild conjunctival infections to serious everlasting ocular damage. Keratopathy (3.6% to 60%), chemosis (9% to 80%), and microbial keratitis are the most prevalent ocular disorders in ICU patients [9]. Insufficient eye care of ICU patients can increase the risk of serious eye problems such as corneal abrasions and ulceration, infectious keratitis, and even corneal perforation and loss of vision [10, 11].

- 1. ***Evidence-based Practice in Eye Care***

In recent decades, many pieces of evidence for eye care were developed; these practices include a wide range of interventions such as 1) washing the eyes with normal saline solution; 2) using lubricating ointments or teardrops; 3) moisturizing the chambers by applying a polyethylene eye cover (PEC), swimming goggles; 4) covering the eyes with eye shields, pads or patch; and 5) closing the eyelid with closure by transparent tape or tarsorrhaphy. There is no clear evidence as to which of these interventions represent the ideal eye care for ICU patients to prevent OSD [3, 4, 8, 10, 12-17]. There is confirmed that the healthcare professionals’ beliefs and traditions have played a major role in selecting eye care interventions in the ICU settings in different countries ICUs [4, 16]. Applying polyethylene eye covers has suggested as the most effective intervention [4] and the main components of the evidence-based protocol to prevent exposure keratopathy in the ICU settings [18]. But, in a very recent study of eye care for intensive care unit, the lubricants and taping of the lids are only advised for the conjunctival and corneal exposure [19]. The PEC was not considered as a part of the standard eye care for critically ill patients as well. So that, the standard care has been only comprised compromised a daily washing of eyelids with normal saline and sterile gauze and application of ocular lubricants at least twice per day in ICUs [20].

- 1. ***Critical Appraisal for Context***

In Iran, washing out eye discharge with sterile normal saline is considered as part of routine eye care for ICU patients, but this intervention is not well supported empirically and is no longer recommended worldwide. Our research team collaborated with health professionals to select alternative eye care interventions to prevent OSD in ICU patients. The team members carefully reviewed empirical and clinical literature to determine the effectiveness or benefits and risks of alternative interventions, while also considering their feasibility in the local ICU contexts. The results of this critical appraisal indicated the following: 1) Irrigating eye with sterile normal saline could cause an increase in the incidence of OSD and cross-infection, and the cotton wool balls can cause corneal scratching [4]. 2) lubrication materials could be hard and time-consuming to apply on a regular or frequent time intervals by nurses [21]; further, the ointment containers can be infected if not used properly. 3) Using eyelid sutures makes regular eye checkups difficult; the sutures are also unpleasant in appearance and block the eye activities [21]. 4) Tarsorrhaphy is also invasive and almost impossible to apply to our lagophthalmos patients. 5) Moisture chamber goggles are only designed for adults and are inconvenient to use when patients are in a supine position [21]. Securing the moisture chamber goggles around the head also increases the risk of edematous eyes in ventilated patients. 6) Mechanical closure of the eyes is recommended in case of the lagophthalmos [22]. 7) Polyethylene covers may be pulled by infants, children or agitated patients [21]. Lastly, eye covering is claimed as the most effective intervention in preventing exposure keratopathy [4].

After examining the benefits and risks of each eye care interventions and its feasibility in our ICU settings, the research team decides to use polyethylene film and artificial teardrops versus normal saline. However, evidence to support their clinical efficacy in reducing the incidence and severity of OSD is limited.

1. **Materials and methods**
   1. ***Aim***

The aim of this is to compare the effects of the polyethylene film and artificial teardrops versus normal saline on the incidence and severity of OSD in comatose patients admitted to the ICU.

- 1. ***Study design***

This is a prospective randomized triple-blinded three-armed clinical trial. We set up three arms ‘A’, ‘B’, and ‘C’ in which by tossing a coin, each eye of a patient will be allocated to one of three interventions: artificial teardrops, polyethylene covers, or normal saline eye drops. The groups will be as the following:

- Group “A”: patients received artificial teardrops for the left eyes and normal saline for the right eyes.
- Group “B”: patients received polyethylene cover for the left eyes and normal saline for the right eyes.
- Group “C”: patients received polyethylene cover to the left eyes and artificial teardrops to the right eyes.

Then, each eligible patient will be allocated to one of the three arms by permuted block randomization.

The study will be approved by the Research and Technology Vice-chancellor after confirming the proposal by the Research Ethics Committee at Hamadan University of Medical Sciences and registering in the Iranian Registry of Clinical Trials. We will conduct the study according to the ethical principles of the Helsinki Declaration of 2013 and follow the CONSORT 2010 statement (http://www.consort-statement.org). The study design is shown in Figure 1.

## Enrollment

Excluded (n= …)

- Not meeting inclusion criteria (n=…)
- Declined to participate (n=…)

**Assessment of eligibility (n=…)**

## Permuted block randomized allocation (n=90)

## (T0)

## Baseline measurement

**Arm** **C** (n=30)

- Received polyethylene cover (Left eye) & artificial teardrops (Right eye)

**Arm** **A** (n=30)

- Received artificial teardrops (Left eye) & normal saline (Right eye).

**Arm** **B** (n=30)

- Received polyethylene cover (Left eye) & normal saline (Right eye)

Analyzed (n=30)

Analyzed (n=30)

## Analysis

## (T1)

## 5 days following T0

Patients completed interventions C **(n=…)**

- Leaving follow-up (= 25%)

Patients completed interventions A **(n=…)**

- Leaving follow-up (= 25%)

**Analyzed (n ≥ 25 patients)**

- Left eyes (n ≥ 25)
- Right eyes (n ≥ 25)

**Analyzed (n ≥ 25 patients)**

- Left eyes (n ≥ 25)
- Right eyes (n ≥ 25)

Patients completed interventions B **(n=…)**

- Leaving to follow-up (= 25%)

## Analysis

**Analyzed (n ≥ 25 patients)**

- Left eyes (n ≥25)
- Right eyes (n ≥ 25)

**Figure 1 CONSORT flow diagram**

- 1. ***Sample size calculation***

The sample size is calculating according to the formula below.

$$n=\left[ \frac{\left( Z_{1-\alpha/2}\sqrt{p_{1}\left( 1-p_{1} \right)} \right)+\left( Z_{1-\beta}\sqrt{p_{2}\left( 1-p_{2} \right)} \right)}{d} \right]^{2}$$

With an 80% power and a 2-sided significance level of α = .05, and the probability of incidence of OSD of a study in which 3.6% (= p_1_) of patients had a positive fluorescein test in the simple eye ointment group and 20.2% (= p_2_) of patients had a positive fluorescein test in the eyelid taping group [5], the number of people needed in each group is 25. Anticipating a total attrition rate of 20%, a total of 90 patients will be included in the study, 30 patients for each arm.

- 1. ***Participants and recruitment***

Patients in the three ICUs in university-affiliated hospitals located in Malayer, Iran, will be recruited:

*2.4.1. Inclusion criteria*

- Newly being admitted to the ICUs;
- Having 18 years of age or older;
- Being comatose (with a Glasgow Coma Scale (GCS) less than or equal to 8 of 15);
- Undergoing mechanical ventilated;
- No trauma in the areas of the face and eyes;
- No history of chronic disease, electrolyte imbalance, cataract surgery or glaucoma;
- Absence of blinking (less than five times per minute); and
- Having a healthy cornea.

*2.4.2. Exclusion criteria*

- Presence of consciousness or natural blinking reflexes;
- Requiring cardiopulmonary resuscitation during the trial;
- Getting discharged from ICUs to the other centers or death.
  1. ***Implementation of the Interventions***

All patients will be received routine eye care. Routine eye care is consisted of eye irrigation with sterile normal saline when necessary (PRN) in all our ICUs. In addition to this usual care, prior to applying the planned interventions, the participants’ eyelids and surrounding skin will be gently irrigated by sterile normal saline and sterile gauze in the same way. The interventions are as below:

- For the intervention with artificial teardrops, while pulling down the lower lid of the patient’s eye to form a V pocket, two Tearlose (Miscellaneous) drops will be applied every six hours.
- For the intervention with normal saline, two drops of saline 0.9% will be applied in the same manner and dose.
- For the intervention with a polyethylene eye cover, 2.5-inch square pieces of thin plastic film will be put from the above of the eyebrow to the cheekbone and fixed by hypoallergenic paper tape.
  1. ***Outcome***

Assessments will be conducted using the statistical software SPSS version 16.0 for windows (SPSS Inc., Chicago, IL), setting the p-values at < 0.05. The measures used and measurements in the study are described in Table 1.

**Table 1. Study measures, source of measurement, and time of assessment.**

| **Construct** | **Questionnaire** | *Assessment times | | |
| --- | --- | --- | --- | --- |
|  |  | **S** | **T0** | **T1** |
| Demographic | Socio-demographic data | * | - | - |
| Clinical characteristics | Clinical history | * | - | - |
| Incidence of Ophthalmic Surface Diseases | The Corneal Fluorescein Staining Pattern | - | * | * |
| Severity of Ophthalmic Surface Diseases | The Corneal Fluorescein Staining Pattern | - | * | * |

*Assessment times: S= Screening, T0= baseline, T1= 5 days after the allocation.

*2.6.1 Primary outcome*

The incidence and severity of OSDs will be assessed with the Corneal Fluorescein Staining Pattern. The cornea will be examined at the slit lamp with a blue filter between four and eight minutes following fluorescein instillation. Punctate epithelial erosions (PEE) will be counted and scored from zero to six, the maximum possible score for each cornea. Grade 0 for no PEE, grade 1 for 1-5 PEE, grade 2 for 6-30 PEE, and grade 3 for more than 30 PEE. A score of severity will be added when the PEE is seen in the central 4mm diameter portion of the cornea, one or more filaments occurred anywhere on the cornea, or one or more patches of confluent staining, including linear stains, if found anywhere on the cornea.

- 1. ***Data Analysis***

In addition to descriptive statistics, the assumption of normality for numerical variables will be examined with the K-S test. Patients’ characteristics will be compared across the three treatment groups, using the Chi-Square test for categorical variables (sex, and type of injury) and one-way ANOVA for numerical variables (age). The incidence of OSD will be compared across the three treatment groups by McNemar’s test and the severity of the OSD by the Kruskal–Wallis test.

1. **Discussion**

This paper describes the evidence-based interventions added to the routine eye care for unconscious patients hospitalized in ICUs. It also presents the study protocol to compare the effectiveness of three methods of eye care, artificial teardrops, polyethylene covers, or normal saline eye drops using a prospective randomized triple-blinded three-armed clinical trial.

Patients who are admitted to Intensive Care Units (ICUs) usually experience failures in one or more vital organ systems such as respiratory, neurologic, and cardiovascular systems accompanied by many other complications which may be resulted in insufficient eye care. Insufficient eye care of ICU patients can increase the risk of serious eye problems such as corneal abrasions and ulceration, infectious keratitis, and even corneal perforation and loss of vision [10, 11]. There are strong agreements upon the existing diversity of the OSD risk factors for patients in ICUs. The comatose patients are suffering from tear film reduction and misaligning over the ocular surface, tear components dilution due to lack of blinking, lid incomplete closing, and lid or conjunctival edema occurring with the use of a ventilator and in the prone position [3-8].

There are many pieces of evidence for eye care which included a wide range of interventions such as eye washing with normal saline solution, using lubricating ointments or teardrops, moisturizing the chambers by applying a polyethylene eye cover (PEC), swimming goggles, and eye shields, closing the eyelid with pads or patch, transparent tape, or tarsorrhaphy. But, there is no clear evidence as to which of these interventions represent the ideal eye care for ICU patients to prevent OSDs [3, 4, 8, 10, 12-17].

Although polyethylene eye covers have been suggested as the most effective intervention[4, 18] the lubricants and taping of the lids are only advised for eye care in the intensive care unit [19]. In many ICUs included ours, the standard care has been only giving in a daily washing of eyelids with normal saline and sterile gauze and application of ocular lubricants at least twice per day [20]. So, there is a large space to generate evidence from high-quality researches to employ the evidence-based nursing practice. This randomized triple blinded three-arm clinical trial can work in this task.

The main strength of this study is to our knowledge, this would be the first randomized clinical trial to examine the incidence and severity of the OSDs among comatose patients in three groups: routine care (Normal saline), evidence-based care (eye-cover and eye lubricant). This study can provide high-quality pieces of evidence for future evidence-based nursing practice. The study limitation would be due to the probability of the attrition rates which been taken into account in the sample size calculation.

1. **Conclusion**

We present the design of our study aimed to promote an evidence-based nursing practice for critically ill patients’ eye care. The results of this study will contribute to the growing research on exploring the best practices. If the expected results are achieved, this could be considered as an important step in the implementation of such nursing care to enhance evidence-based practice in critically ill patients to prevent OSDs.

**References**

1. Oh EG, Lee WH, Yoo JS, Kim SS, Ko IS, Chu SH, et al. Factors related to incidence of eye disorders in Korean patients at intensive care units. Journal of Clinical Nursing. 2009;18(1):29-35. doi: <http://doi.org/10.1111/j.1365-2702.2008.02388.x>.

2. Werli-Alvarenga A, Ercole FF, Botoni FA, Oliveira JADMM, Chianca TCM. Corneal injuries: incidence and risk factors in the Intensive Care Unit. Revista latino-americana de enfermagem. 2011;19(5):1088-95.

3. de França CFSM, de Lima Fernandes APN, Pinto DPdSR, de Mesquita Xavier SS, Júnior MAF, Botarelli FR, et al. Evidence of interventions for the risk of dry eye in critically ill patients: An integrative review. Applied Nursing Research. 2016;29:e14-e7. doi: <http://doi.org/10.1016/j.apnr.2015.05.016>.

4. Alansari MA, Hijazi MH, Maghrabi KA. Making a difference in eye care of the critically ill patients. Journal of Intensive Care Medicine. 2015;30(6):311-7. doi: <http://doi.org10.1177/0885066613510674>.

5. Ahmadi-Nejad M, Ranjbar H, Karbasi N, Borhani F, Karzari Z, Moghaddar M. [Comparing the effectiveness of two methods of eye care in the prevention of ocular surface disorders in patients hospitalized in intensive care unit]. Annals of Military and Health Sciences Research. 2013;10(4):323-8.

6. Ghanei M, Matin S, Radmehr M, Pakdel M, Kalani N. Comparison of three methods of wet gauze, adhesive tape and eye ointment to prevent corneal ulceration in pationts undergoing general anestesia. Journal of Fundamental and Applied Sciences. 2016;8(2S):16-27.

7. Demirel S, Cumurcu T, Fırat P, Aydogan MS, Doğanay S. Effective management of exposure keratopathy developed in intensive care units: The impact of an evidence based eye care education programme. Intensive and Critical Care Nursing. 2014;30(1):38-44. doi: <http://doi.org/10.1016/j.iccn.2013.08.001>.

8. Oliveira RS, Fernandes APNdL, Botarelli FR, Araújo JNdM, Barreto VP, Vitor AF. Risk factors for injury in the cornea in critical patients in intensive care: an integrative review. Revista de Pesquisa: Cuidado é Fundamental Online. 2016;8(2):4423-34.

9. Grixti A, Sadri M, Edgar J, Datta AV. Common ocular surface disorders in patients in intensive care units. The Ocular Surface. 2012;10(1):26-42. doi: <http://doi.org/10.1016/j.jtos.2011.10.001>.

10. Rosenberg JB, Eisen LA. Eye care in the intensive care unit: Narrative review and meta-analysis. Critical Care Medicine. 2008;36(12):3151-5. doi: <http://doi.org/10.1097/CCM.0b013e31818f0ee7>.

11. Saritas TB, Bozkurt B, Simsek B, Cakmak Z, Ozdemir M, Yosunkaya A. Ocular surface disorders in intensive care unit patients. The Scientific World Journal. 2013;2013(182038). doi: <http://doi.org/10.1155/2013/182038>.

12. Taheri-Kharameh Z. 177: Eye care in the intensive care patients: An evidence based review. BMJ Open. 2017;7(Suppl 1):bmjopen-2016-015415.177. doi: <http://doi.org/10.1136/bmjopen-2016-015415.177>.

13. Sivasankar S, Jasper S, Simon S, Jacob P, John G, Raju R. Eye care in ICU. Indian Journal of Critical Care Medicine. 2006;10(1):11.

14. Marshall AP, Elliott R, Rolls K, Schacht S, Boyle M. Eyecare in the critically ill: Clinical practice guideline. Australian Critical Care. 2008;21(2):97-109. doi: <http://doi.org/10.1016/j.aucc.2007.10.002>.

15. Hillier S, Grimmer-Somers K, Merlin T, Middleton P, Salisbury J, Tooher R, et al. FORM: an Australian method for formulating and grading recommendations in evidence-based clinical guidelines. BMC Medical Research Methodology. 2011;11(1):23. doi: <http://doi.org/10.1186/1471-2288-11-23>.

16. Guler EK, Eser I, Fashafsheh IHD. Intensive care nurses' views and practices for eye care: An international comparison. Clin Nurs Res. 2017;26(4):504-24. Epub 2016/02/20. doi: <http://doi.org/10.1177/1054773816631471>. PubMed PMID: 26893447.

17. Hearne BJ, Hearne EG, Montgomery H, Lightman SL. Eye care in the intensive care unit. Journal of the Intensive Care Society. 2018:1751143718764529. doi: <http://doi.org/10.1177/1751143718764529>.

18. Yim W-y. Evidence-based eye care protocol for ICU patients with altered level of consciousness. HKU Theses Online (HKUTO). 2009.

19. Hearne BJ, Hearne EG, Montgomery H, Lightman SL. Eye care in the intensive care unit. Journal of the Intensive Care Society. 2018;19(4):345–50. doi: <http://doi.org/10.1177/1751143718764529>.

20. Bates J, Dwyer R, O'Toole L, Kevin L, O'Hegarty N, Logan P. Corneal protection in critically ill patients: a randomized controlled trial of three methods. Clinical Intensive Care. 2004;15(1):23-6. . doi: <http://doi.org/10.5539/gjhs.v8n7p212>.

21. Sharjeel M, Malik IQ, Iqbal CJ, Ali F. Prevention of exposure keratopathy with sahaf wet chamber. Pakistan Journal of Ophthalmology. 2015;31(3):131-6.

22. Van der Wekken R, Torn E, Ros F, Haas L. A red eye on the intensive care unit. Exposure keratopathy with corneal abrasion secondary to lagophthalmos due to chemosis. Neth J Med. 2013;71(4):204-7.
