# Supplementary material for: Polyethylene eye-cover versus artificial teardrops in the prevention of ocular surface diseases in comatose patients: A prospective multicenter randomized triple-blinded three-arm clinical trial
Source: PLoS One. 2021 Apr 1;16(4):e0248830. doi: 10.1371/journal.pone.0248830 (PMC8016328; doi:10.1371/journal.pone.0248830)
Supplement: S2 Protocol — (DOCX) [file pone.0248830.s015.docx]

**تاثیر کاور پلی اتیلن و قطره اشک مصنوعی نسبت به نرمال سالین بر بروز اختلالات سطحی چشم در بیماران غیرهوشیار: کارآزمایی بالینی آینده نگر تصادفی شده سه گروهی سه سو کور**

**مقدمه:** ادعا می شود که کاور پلی اتیلن در جلوگیری از بیماری های سطح چشم (OSD) مفید هستند. با این حال، شواهد در مورد اثربخشی بالینی آنها محدود است. این کارآزمایی بالینی با هدف مقایسه تأثیر کاور پلی اتیلن و اشک مصنوعی در مقابل سالین نرمال بر بروز و شدت OSD در بیماران بیهوش انجام شده است. این مقاله خلاصه پروتکل مطالعه می باشد.

**روش**: مطالعه پیش رو، یک کارآزمایی بالینی با طرح تصادفی سه گروهی سه سو کور است. طبق محاسبه، در هر گروه به 25 بیمار نیاز است، اما با احتساب 20٪ ریزش، در هر گروه 30 بیمار و درمجموع 90 بیمار انتخاب می شوند. ابتدا با پرتاب سکه برای هر یک از چشم های بیمار هر گروه "الف"، "ب" یا "ج" ، یکی از سه مداخله اشک مصنوعی، کاور پلی اتیلن، یا قطره نرمال سالین تعیین خواهد شد. سپس، هر بیمار واجد شرایط با تصادفی سازی بلوک جایگشتی به یکی از سه بازو اختصاص می یابد. همه بیماران نبیهوش هستند لذا کورسازی در مورد آنان صدق نمی کند. هم فرد معاینه کننده چشم بیماران و هم تحلیلگر آماری از نوع مداخلات آگاهی ندارند. چشمان بیماران بر اساس فرم درجه بندی شدت اختلالات سطحی چشم بررسی خواهد شد. داده های جمع آوری شده توسط نرم افزار SPSS-16 در سطح اطمینان 95٪ تجزیه و تحلیل خواهد شد.

**بحث و نتيجه گيري**: نتايج اين مطالعه به توسعه شواهدی متقن جهت ارائه يك مداخله مراقبت چشمي بي خطر ، مؤثر و در دسترس براي جلوگيري از بروز OSD در بيماران کمایی بستری در بخش های مراقبت ویژه كمك مي كند.

**کلیدواژه ها**: بخش های مراقبت ویژه؛ بیماران بستری؛ بیماری های عفونی؛ چشم؛ کما؛ مراقبت پرستاری

1. **مقدمه**

هر چند اختلالات سطحی چشم^[[1]](#footnote-1)^ (OSD) عارضه ای شایع در بیمارانی است که در بخش مراقبت های ویژه (ICU) بستری می شوند اما برای جلوگیری از بروز آن حداقل توجه مراقبتی را دریافت می کنند[1, 2].. OSD با عوامل خطر مختلفی همراه است، از جمله اختلال در ترشح اشکی و توزیع آن بر روی سطح چشم، رقیق شدن غلظت اشک به دلیل عدم رفلکس پلک زدن، بسته شدن ناقص پلک ها^[[2]](#footnote-2)^، یا ادم ملتحمه که به دلیل استفاده از دستگاه تهویه کننده به وجود می آید [3-8]. طیف وسیعی از عوارض چشم از عفونت ملتحمه ملایم تا آسیب جدی مادام العمر چشمی برای این بیماران وجود دارد. کراتوپاتی^[[3]](#footnote-3)^ (6/3٪ تا 60٪) ، کیموز^[[4]](#footnote-4)^ (9٪ تا 80٪) و کراتیت میکروبی شایعترین اختلالات چشمی در بیماران ICU می باشند[9]. عدم مراقبت کافی از چشم در بیماران ICU می تواند خطر بروز مشکلات جدی چشم مانند ساییدگی و زخم قرنیه، کراتیت عفونی و حتی سوراخ شدن قرنیه و از بین رفتن بینایی را افزایش دهد[10, 11].

- 1. ***عملکرد مبتنی بر شواهد^[[5]](#footnote-5)^ در مراقبت از چشم***

در دهه های اخیر ، شواهد بسیاری برای مراقبت از چشم ارائه شده است. این روشها طیف گسترده ای از مداخلات را در بر می گیرند مانند: 1) شستشوی چشم با محلول نرمال سالین، 2) استفاده از پمادهای لوبریکنت یا قطره اشک مصنوعی، 3) مرطوب نگهداشتن چشم با کاور پلی اتیلن یا عینک شنا، 4) پوشاندن چشم با شیلد محافظ یا پد یا گاز، و 5) بستن پلک با استفاده از چسب ضد حساسیت شفاف یا دوختن پلک ها^[[6]](#footnote-6)^. تا کنون شواهد مشخصی وجود ندارد که نشان دهد کدام یک از این مداخلات جهت پیشگیری از ابتلا به اختلالات سطحی چشم در بیماران بستری در بخش مراقبت های ویژه مطلوب می باشد [3, 4, 8, 10, 12-17]. تأیید شده است که اعتقادات و سنت های اعضای تیم های مراقبت سلامت بر انتخاب روش مراقبت از چشم در محیط های بالینی ICU در کشورهای مختلف نقش عمده ای دارد[4, 16].

استفاده از پوشش های پلی اتیلن چشم به عنوان مؤثرترین مداخله [4] و مؤلفه های اصلی پروتکل مبتنی بر شواهد برای جلوگیری از قرار گرفتن در معرض کراتوپاتی در محیط های ICU پیشنهاد شده است [18]. اما ، در یک مطالعه اخیر در مورد مراقبت از چشم در ICU فقط استفاده از لوبریکنت و چسب زدن پلک برای جلوگیری از خشکی ملتحمه و قرنیه توصیه شده است[19]. و استفاده از کاور پلی اتیلن چشم به عنوان مراقبت های استاندارد چشم برای بیماران بحرانی در نظر گرفته نشده است. یعنی، مراقبت استاندارد چشم در ICU فقط شستشوی روزانه پلك ها با گاز استریل آغشته به نرمال سالین و پمادهای چشمی حداقل دو بار در روز را شامل می شود [20].

- 1. ***ارزیابی نقادانه بر اساس محیط پژوهش***

در ایران، شستشوی چشم با محلول نرمال سالین استریل بخشی از مراقبت معمول چشم برای بیماران ICU در نظر گرفته می شود، اما این روش از نظر تجربی مطلوب نیست و دیگر در سراسر جهان توصیه نمی شود. تیم پژوهشی ما با همکاری افراد متخصص مراقبتی- درمانی برای پیشگیری از ابتلا به OSD اقدام به انتخاب روش مراقبتی مناسب برای جایگزین کردن مراقبت روتین چشم بیماران بستری در ICU کردند. اعضای این تیم برای تعیین اثربخشی یا مزایا و خطرات مداخله جایگزین، متون پژوهشی و بالینی را با دقت مرور و براساس امکان سنجی اجرای آنها در ICU ارزیابی نقادانه نمودند. نتایج این ارزیابی نقادانه بیانگر موارد زیر است: 1) شستشوی چشم با محلول نرمال سالین استریل می تواند باعث انتقال عفونت و افزایش شیوع OSD شود و استفاده از گاز یا سوآپ پنبه ای می تواند باعث خراش قرنیه شود[4]. 2) استفاده از پماد یا قطره لوبریکنت چشمی در فواصل زمانی منظم و مکرر توسط پرستاران می تواند سخت و وقت گیر باشد [21]. علاوه بر این، پماد و یا قطره اگر به درستی استفاده نشوند، می توانند آلوده شوند؛ 3) استفاده از بخیه جهت تراسورافی پلک ها نه تنها معاینه منظم چشم را دشوار می کند بلکه از نظر ظاهری ناخوشایند هستند و جلوی حرکات چشم را می گیرد [21]. 4) همچنین تراسورافی یک روش تهاجمی محسوب می شود و برای تمام بیماران دچار لاگوفتالموس مناسب نیست. 5) استفاده از عینک جهت حفظ رطوبت چشم فقط برای بزرگسالان امکان پذیر است و استفاده از آن برای بیمار در وضعیت طاقباز مشکل است[21]. همچنین بستن عینک به دور سر بیمار باعث افزایش خطر ادم چشم بیماران تحت ونتیلاتور می شود. بستن چشم ها در مواردی که لاگوفتالموس وجود دارد توصیه شده است [22]. کاورهای پلی اتیلن نیز ممکن است توسط شیرخواران، کودکان و بیماران آژیته کشیده و پاره شوند [21]. در نهایت، تایید شده است که کاور چشم ها موثرترین روش در پیشگیری از خشکی قرنیه تماسی^[[7]](#footnote-7)^ می باشد [4].

تیم تحقیق پس از بررسی مزایا و خطرات هر یک از روش های مراقبت از چشم و امکان سنجی آن براساس محیط و امکاناتICU ما، تصمیم به استفاده از کاور پلی اتیلن و اشک مصنوعی در مقابل نرمال سالین گرفت. هرچند که شواهدی برای حمایت از اثربخشی بالینی آنها در کاهش بروز و شدت OSD محدود است.

1. **روش**
   1. ***هدف***

هدف از پژوهش حاضر تعیین تاثیر کاور پلی اتیلن و قطره اشک مصنوعی نسبت به نرمال سالین بر بروز و شدت اختلالات سطحی چشم در بیماران غیرهوشیار است.

- 1. ***نوع*** ***مطالعه***

مطالعه حاضر یک کارآزمایی بالینی تصادفی آینده نگر سه گروهی سه سو کور است. ما سه گروه "A" ، "B" و "C" را در نظر گرفته ایم که در آن با پرتاب یک سکه هر چشم بیمار به یکی از سه مداخله تخصیص می یابد: قطره اشک مصنوعی، کاور پلی اتیلن یا قطره نرمال سالین چشم. گروه ها به شرح زیر خواهند بود:

- گروه (A چشم چپ قطره اشک مصنوعی+چشم راست سرم نرمال سالين
- گروه (B چشم چپ كاور پلي اتيلن+چشم راست سرم نرمال سالين
- گروه (C چشم چپ كاور پلي اتيلن+چشم راست قطره اشک مصنوعی

سپس ، هر بیمار واجد شرایط با تصادفی سازی بلوک جایگشتی به یکی از سه گروه اختصاص خواهد یافت.

این مطالعه پس از تأیید توسط کمیته اخلاق تحقیقات دانشگاه علوم پزشکی همدان و تایید توسط مرکز ثبت کارآزمایی های بالینی ایران، توسط معاونت تحقیقات و فناوری تصویب خواهد شد. ما این مطالعه را طبق اصول اخلاقی اعلامیه هلسینکی سال 2013 انجام خواهیم داد و بیانیه CONSORT 2010 (http://www.consort-statement.org) را دنبال خواهیم کرد. طرح مطالعه در شکل 1 نشان داده شده است.

## ثبت نام بیماران

**ریزش** (n= …)

- نداشتن معیار ورود (n=…)
- عدم تمایل به مشارکت (n=…)

**بررسی بیماران واجد شرایط (n=…)**

## (T0)

## بدو ورود به مطالعه

## تخصیص تصادفی با روش بلوک جایگشتی (n=90)

**گروه** **A** (n=30)

- چشم چپ قطره اشک مصنوعی+چشم راست سرم نرمال سالين

**گروه** **B** (n=30)

- چشم چپ كاور پلي اتيلن+چشم راست سرم نرمال سالين

**گروه** **C** (n=30)

- چشم چپ كاور پلي اتيلن+چشم راست قطره اشک مصنوعی

تعداد بیمارانی که مداخله را کامل کرده اند C **(n=…)**

- ریزش نمونه (25%=)

تعداد بیمارانی که مداخله را کامل کرده اندA **(n=…)**

- ریزش نمونه(25%=)

تعداد بیمارانی که مداخله را کامل کرده اندB **(n=…)**

- ریزش نمونه (25%=)

## (T1)

## روز پنجم

Analyzed (n=30)

Analyzed (n=30)

## Analysis

## تحلیل

**تعداد بیماران تحلیل (n ≥ 25 patients)**

- چشم چپ (n ≥ 25)
- چشم راست (n ≥ 25)

**تعداد بیماران تحلیل (n ≥ 25 patients)**

- چشم چپ (n ≥ 25)
- چشم راست (n ≥ 25)

**تعداد بیماران تحلیل (n ≥ 25 patients)**

- چشم چپ (n ≥ 25)
- چشم راست (n ≥ 25)

**شکل شماره 1 فلوچارت CONSORT**

- 1. ***محاسبه حجم نمونه***

تعداد افراد هرگروه براساس فرمول زیر محاسبه شد.

$$n=\left[ \frac{\left( Z_{1-\alpha/2}\sqrt{p_{1}\left( 1-p_{1} \right)} \right)+\left( Z_{1-\beta}\sqrt{p_{2}\left( 1-p_{2} \right)} \right)}{d} \right]^{2}$$

با در نظر گرفتن توان آزمون 80٪ و سطح معناداری دو طرفه α = 0.05 و احتمال بروز OSD دریک مطالعه مداخله ای که دو روش مراقبت چشم را مقایسه کرده است،احتمال 6/3٪ (= p1) بیماران مثبت در گروه پماد چشم و احتمال 2/20٪ (= P2) در بیماران مثبت در گروه بستن پلك ها [5]، تعداد افراد مورد نیاز در هر گروه 25 نفر به دست آمده است. با پیش بینی كل میزان ریزش 25٪ ، در مجموع 90 بیمار (30 بیمار برای هر گروه) نمونه گیری خواهند شد.

- 1. ***نمونه گیری***

بیماران در سه بخش مراقبت های ویژه در بیمارستان های واقع در ملایر، ایران در مطالعه شرکت داده خواهند شد:

*2-4-1 معیارهای ورود*

- بستری شدن جدید در ICU؛
- داشتن 18 سال یا بالاتر؛
- داشتن وضعیت کما (سطح هوشیاری 8 و پایین تر برحسب معیار گلاسکو کما)؛
- تحت ونتیلاتور (تهویه مکانیکی)؛
- عدم ترومای مستقیم به ناحیه صورت و چشم ها؛
- نداشتن سابقه عمل جراحی کاتاراکت و نداشتن بیماری گلوکوم با زاویه باز یا بسته؛
- عدم وجود یا تضعیف رفلکس پلک زدن، طبق تایید متخصص محترم چشم ( کمتر از پنج بار در دقیقه)؛
- سلامت اولیه قرنیه ( با تایید متخصص محترم چشم با استفاده از کاغذ فلورسین و پس از معاینه با اسلیت لامپ پرتابل).

*2-4-1 معیارهای ورود*

- افزایش هوشیاری یا بازگشت رفلکس پلک زدن؛
- نیاز به عملیات احیاء قلبی ریوی؛
- ترخيص، انتقال از بخش، اعزام به مراکز دیگر يا فوت بيمار.
  1. ***نحوه مداخله***

از همه بیماران مراقبت روتین از چشم انجام خواهد شد. مراقبت روتین چشم عبارت از شستشوی چشم با سالین نرمال استریل در صورت لزوم (PRN) در تمام ICU مورد نظر ما می باشد. علاوه بر این مراقبت روتین، قبل از اعمال مداخلات برنامه ریزی شده در این مطالعه، پلک های شرکت کنندگان و پوست اطراف آن به آرامی توسط سالین نرمال و گاز استریل به آرامی شستشو داده میشود. سپس مداخلات به شرح زیر انجام می شود:

- برای مداخله با قطره اشک های مصنوعی، دو قطره Tearlose (Miscellaneous) در کیسه V شکل حاصل از پایین کشیدن پلک پایین چشم بیمار هر شش ساعت یکبار چکانده می شود.
- برای مداخله با نرمال سالین ، دو قطره نرمال سالین 9/0٪ به همان روش بالا هر شش ساعت یکبار چکانده می شود.
- برای مداخله با کاور چشمی پلی اتیلن، قطعات مربع 5/2 اینچی از نایلون پلاستیکی نازک چیده و از بالای ابرو تا گونه توسط چسب نواری هیپوالرژیک ثابت می شوند.
  1. ***پیامد مداخله***

داده های گردآوری شده با استفاده از نرم افزار SPSS نسخه 16 در سطح خطای 05/0 مورد تحلیل قرار خواهد گرفت. آزمون ها و زمان های ارزیابی در جدول 1 نشان داده شده است.

**جدول1. متغیرها، ابزارها و زمان های اندازه گیری در مطالعه**

| متغیر | پرسشنامه | غربالگری | T0  بدو ورود به مطالعه | T1  پنج روز بعد |
| --- | --- | --- | --- | --- |
| اطلاعات فردی | ویژگی های فردی | * | - | - |
| اطلاعات بالینی | تاریخچه سلامتی | * | - | - |
| بروز اختلالات سطحی چشم | فرم درجه بندی شدت اختلالات سطحی چشم | - | * | * |
| شدت اختلالات سطحی چشم | فرم درجه بندی شدت اختلالات سطحی چشم | - | * | * |

*2-6-1 پیامد اولیه*

بروز و شدت OSDs با استفاده از اسلیت لامپ پرتابل و 8-4 دقیقه پس از استفاده از کاغذ فلورسین توسط متخصص چشم مورد معاینه و براساس فرم درجه بندی شدت اختلالات سطحی^[[8]](#footnote-8)^ از لحاظ زخم نفوذی اپیتلیال (PEE)^[[9]](#footnote-9)^، چشم نمره دهی خواهد شد. این فرم سطح بندی شامل درجه بندی از صفر تا 6 می باشد: درجه صفر نشان دهنده عدم بروز PEE، درجه 1 برای 5-1 تا PEE و درجه 2 برای 30-6 PEE و درجه 3 برای بیش از 30 PEE در قرنیه چشم لحاظ می شود. در صورت وجود هر یک از موارد زیر، یک نمره جهت شدت زخم نفوذی اپیتلیال به نمرات فوق اضافه می شد. هنگامی که PEE به اندازه 4 میلی متر در مرکز قرنیه دیده شود، حداقل یک کدورت رشته ای در هر کجای قرنیه دیده شود، و یک یا چند کدورت لکه ای یا رشته ای در هر کجای قرنیه دیده شود. در این مطالعه کسب نمره صفر به معنی عدم وجود اختلال سطحی چشم و کسب نمره یک و بالاتر به معنی بروز اختلال سطحی چشم تلقی گردید.

- 1. ***تحلیل داده ها***

علاوه بر آمار توصیفی، جهت بررسی نرمال بودن توزیع داده ها از آزمون کولموگروف- اسمیرونف استفاده خواهد شد. ویژگی های فردی و بالینی شرکت کنندگان با استفاده از آزمون مجذور کای برای داده های اسمی (جنس، نوع بیماری) و از آزمون آنالیز واریانس یکطرفه برای داده های عددی (سن) استفاده خواهد شد. بروز OSD بین سه گروه با آزمون مک نمار و شدت OSD با آزمون کروسکال-والیس مورد بررسی قرار خواهد گرفت.

1. **بحث**

در این مطالعه علاوه بر مراقبت چشم روتین، مداخلات مبتنی بر شواهدی را که برای بیماران کمایی بستری در بخش های مراقبت ویژه انجام خواهد شد، توصیف شده است. همچنین در این مقاله، پروتکل مطالعه جهت مقایسه اثربخشی سه روش مراقبت از چشم (قطره اشک مصنوعی، کاور پلی اتیلن یا قطره سالین نرمال چشم) را با استفاده از یک کارآزمایی بالینی آینده نگر تصادفی سه سو-کور ارائه می کند.

بیمارانی که در ICU بستری هستند، معمولاً در یک یا چند سیستم ارگانیسم حیاتی مانند سیستم تنفسی، قلبی-عروقی، کلیوی دچار نارسایی می شوند که ممکن است منجر به مراقبت کافی از چشم شوند. عدم مراقبت کافی از چشم در بیماران ICU می تواند خطر بروز مشکلات جدی چشم مانند ساییدگی و زخم قرنیه، کراتیت عفونی و حتی سوراخ شدن قرنیه و از بین رفتن بینایی را افزایش دهد[10, 11]. توافق گسترده ای در مورد وجود انواع عوامل خطر OSD برای بیماران در بخش های مراقبت ویژه وجود دارد. بیماران کومایی از کاهش ترشح و پخش در سطح چشمی، رقیق شدن غلظت اشک به دلیل عدم پلک زدن، بسته شدن ناقص پلک، ادم پلک و یا ملتحمه که به دلیل استفاده از دستگاه تهویه یا قرارگیری در پوزیشن دمر^[[10]](#footnote-10)^، رنج می برند[3-8].

شواهد زیادی برای مراقبت از چشم وجود دارد که شامل طیف گسترده ای از مداخلات مانند شستشوی چشم با محلول نرمال سالین، استفاده از پمادهای روان کننده یا اشک مصنوعی، حفظ رطوبت چشم با استفاده از یک پوشش چشم پلی اتیلن (PEC)، عینک شنا و محافظ چشم است. بسته شدن پلک با پد یا پانسمان، چسب نواری شفاف یا بخیه زدن می باشد. اما، هیچ شواهد روشنی وجود ندارد که نشان دهد کدام یک از این مداخلات، مراقبتی ایده آل از چشم بیماران بستری در بخش مراقبت های ویژه برای پیشگیری از ابتلا به OSD فراهم می آورد[3, 4, 8, 10, 12-17].

اگرچه کاور پلی اتیلن چشم به عنوان مؤثرترین مداخله پیشنهاد شده است[4, 18]، هنوز هم لوبریکنت و چسب زدن به پلک بیماران ICU برای مراقبت از چشم توصیه می شود[19]. در بسیاری از ICU های از جمله بخش های مراقبت ویژه محیط پژوهش، مراقبت استاندارد فقط در شستشوی روزانه پلک ها با سالین نرمال و گاز استریل و استفاده از لوبریکنت های چشمی حداقل دو بار در روز انجام می شود[20]. بنابراین، فضای زیادی برای تولید شواهد حاصل از تحقیقات با کیفیت بالا برای استفاده از روش پرستاری مبتنی بر شواهد وجود دارد. این کارآزمایی بالینی آینده نگر تصادفی سه سو-کور سه گروهی می تواند در این امر مؤثر باشد.

براساس مطالعات ما، نقطه قوت این مطالعه این است که این اولین کارآزمایی بالینی تصادفی است که به بررسی شیوع و شدت OSD در بیماران کمایی در سه گروه می پردازد: مراقبت روزمره (نرمال سالین) ، مراقبت مبتنی بر شواهد (پوشش چشم و قطره لوبریکنت چشمی). این مطالعه می تواند به ارائه شواهد با کیفیت بالا برای اقدامات پرستاری مبتنی بر شواهد در آینده کمک نماید. محدودیت مطالعه به دلیل احتمال ریزش نمونه است که در محاسبه اندازه نمونه در نظر گرفته شده است.

1. **نتیجه گیری**

مطالعه حاضر با هدف ترویج پرستاری مبتنی بر شواهد برای مراقبت از چشم بیماران ICU ارائه شده است. نتایج این تحقیق به رشد روزافزون تحقیقات در مورد کشف بهترین شیوه های مراقبتی کمک خواهد کرد. در صورت دستیابی به نتایج مورد انتظار، این مطالعه می تواند به عنوان یک گام مهم در اجرای چنین مراقبت های پرستاری در جهت تقویت عمل مبتنی بر شواهد در بیماران بحرانی در نظر گرفته شود تا از ابتلا به OSD پیشگیری شود.

**منابع**

1. Oh EG, Lee WH, Yoo JS, Kim SS, Ko IS, Chu SH, et al. Factors related to incidence of eye disorders in Korean patients at intensive care units. Journal of Clinical Nursing. 2009;18(1):29-35. doi: <http://doi.org/10.1111/j.1365-2702.2008.02388.x>.

2. Werli-Alvarenga A, Ercole FF, Botoni FA, Oliveira JADMM, Chianca TCM. Corneal injuries: incidence and risk factors in the Intensive Care Unit. Revista latino-americana de enfermagem. 2011;19(5):1088-95.

3. de França CFSM, de Lima Fernandes APN, Pinto DPdSR, de Mesquita Xavier SS, Júnior MAF, Botarelli FR, et al. Evidence of interventions for the risk of dry eye in critically ill patients: An integrative review. Applied Nursing Research. 2016;29:e14-e7. doi: <http://doi.org/10.1016/j.apnr.2015.05.016>.

4. Alansari MA, Hijazi MH, Maghrabi KA. Making a difference in eye care of the critically ill patients. Journal of Intensive Care Medicine. 2015;30(6):311-7. doi: <http://doi.org10.1177/0885066613510674>.

5. Ahmadi-Nejad M, Ranjbar H, Karbasi N, Borhani F, Karzari Z, Moghaddar M. [Comparing the effectiveness of two methods of eye care in the prevention of ocular surface disorders in patients hospitalized in intensive care unit]. Annals of Military and Health Sciences Research. 2013;10(4):323-8.

6. Ghanei M, Matin S, Radmehr M, Pakdel M, Kalani N. Comparison of three methods of wet gauze, adhesive tape and eye ointment to prevent corneal ulceration in pationts undergoing general anestesia. Journal of Fundamental and Applied Sciences. 2016;8(2S):16-27.

7. Demirel S, Cumurcu T, Fırat P, Aydogan MS, Doğanay S. Effective management of exposure keratopathy developed in intensive care units: The impact of an evidence based eye care education programme. Intensive and Critical Care Nursing. 2014;30(1):38-44. doi: <http://doi.org/10.1016/j.iccn.2013.08.001>.

8. Oliveira RS, Fernandes APNdL, Botarelli FR, Araújo JNdM, Barreto VP, Vitor AF. Risk factors for injury in the cornea in critical patients in intensive care: an integrative review. Revista de Pesquisa: Cuidado é Fundamental Online. 2016;8(2):4423-34.

9. Grixti A, Sadri M, Edgar J, Datta AV. Common ocular surface disorders in patients in intensive care units. The Ocular Surface. 2012;10(1):26-42. doi: <http://doi.org/10.1016/j.jtos.2011.10.001>.

10. Rosenberg JB, Eisen LA. Eye care in the intensive care unit: Narrative review and meta-analysis. Critical Care Medicine. 2008;36(12):3151-5. doi: <http://doi.org/10.1097/CCM.0b013e31818f0ee7>.

11. Saritas TB, Bozkurt B, Simsek B, Cakmak Z, Ozdemir M, Yosunkaya A. Ocular surface disorders in intensive care unit patients. The Scientific World Journal. 2013;2013(182038). doi: <http://doi.org/10.1155/2013/182038>.

12. Taheri-Kharameh Z. 177: Eye care in the intensive care patients: An evidence based review. BMJ Open. 2017;7(Suppl 1):bmjopen-2016-015415.177. doi: <http://doi.org/10.1136/bmjopen-2016-015415.177>.

13. Sivasankar S, Jasper S, Simon S, Jacob P, John G, Raju R. Eye care in ICU. Indian Journal of Critical Care Medicine. 2006;10(1):11.

14. Marshall AP, Elliott R, Rolls K, Schacht S, Boyle M. Eyecare in the critically ill: Clinical practice guideline. Australian Critical Care. 2008;21(2):97-109. doi: <http://doi.org/10.1016/j.aucc.2007.10.002>.

15. Hillier S, Grimmer-Somers K, Merlin T, Middleton P, Salisbury J, Tooher R, et al. FORM: an Australian method for formulating and grading recommendations in evidence-based clinical guidelines. BMC Medical Research Methodology. 2011;11(1):23. doi: <http://doi.org/10.1186/1471-2288-11-23>.

16. Guler EK, Eser I, Fashafsheh IHD. Intensive care nurses' views and practices for eye care: An international comparison. Clin Nurs Res. 2017;26(4):504-24. Epub 2016/02/20. doi: <http://doi.org/10.1177/1054773816631471>. PubMed PMID: 26893447.

17. Hearne BJ, Hearne EG, Montgomery H, Lightman SL. Eye care in the intensive care unit. Journal of the Intensive Care Society. 2018:1751143718764529. doi: <http://doi.org/10.1177/1751143718764529>.

18. Yim W-y. Evidence-based eye care protocol for ICU patients with altered level of consciousness. HKU Theses Online (HKUTO). 2009.

19. Hearne BJ, Hearne EG, Montgomery H, Lightman SL. Eye care in the intensive care unit. Journal of the Intensive Care Society. 2018;19(4):345–50. doi: <http://doi.org/10.1177/1751143718764529>.

20. Bates J, Dwyer R, O'Toole L, Kevin L, O'Hegarty N, Logan P. Corneal protection in critically ill patients: a randomized controlled trial of three methods. Clinical Intensive Care. 2004;15(1):23-6. . doi: <http://doi.org/10.5539/gjhs.v8n7p212>.

21. Sharjeel M, Malik IQ, Iqbal CJ, Ali F. Prevention of exposure keratopathy with sahaf wet chamber. Pakistan Journal of Ophthalmology. 2015;31(3):131-6.

22. Van der Wekken R, Torn E, Ros F, Haas L. A red eye on the intensive care unit. Exposure keratopathy with corneal abrasion secondary to lagophthalmos due to chemosis. Neth J Med. 2013;71(4):204-7.

1. Ocular Surface Disease [↑](#footnote-ref-1)
2. Lagophthalmos [↑](#footnote-ref-2)
3. Keratopathy [↑](#footnote-ref-3)
4. Chemosis [↑](#footnote-ref-4)
5. Evidence-based Practice [↑](#footnote-ref-5)
6. Tarsorrhaphy [↑](#footnote-ref-6)
7. Exposure keratopathy [↑](#footnote-ref-7)
8. Corneal Fluorescein Staining Pattern [↑](#footnote-ref-8)
9. Punctate epithelial erosions (PEE) [↑](#footnote-ref-9)
10. Prone position [↑](#footnote-ref-10)
