# Supplementary material for: Polyethylene eye-cover versus artificial teardrops in the prevention of ocular surface diseases in comatose patients: A prospective multicenter randomized triple-blinded three-arm clinical trial
Source: PLoS One. 2021 Apr 1;16(4):e0248830. doi: 10.1371/journal.pone.0248830 (PMC8016328; doi:10.1371/journal.pone.0248830)
Supplement: Attachment — Submitted filename: WITHOUT TABLE Response to reviewers - Copy.docx [file pone.0248830.s016.docx]

Dear Editors and Reviewers,

Thank you for your email and for the reviewers' comments concerning our manuscript entitled  ”Polyethylene eye cover versus artificial teardrops in the prevention of ocular surface diseases in comatose patients: a prospective multicenter randomized triple-blinded three-arm clinical trial” no: PONE-D-19-33881. Those comments are all valuable and very helpful for re-revising and improving our manuscript, as well as the important guiding significance to our researches. We have studied comments carefully and have made revised which we hope meet with approval. Revised portion are highlighted in the manuscript. The main corrections in the paper and the responds to the Editor’s and reviewer’s comments are as flowing:

Journal Requirements:

When submitting your revision, we need you to address these additional requirements.

1. Please ensure that your manuscript meets PLOS ONE's style requirements, including those for file naming. The PLOS ONE style templates can be found at https://journals.plos.org/plosone/s/file?id=wjVg/PLOSOne_formatting_sample_main_body.pdf and https://journals.plos.org/plosone/s/file?id=ba62/PLOSOne_formatting_sample_title_authors_affiliations.pdf

**Response:** My manuscript comes across PLOS ONE's style requirements.

2. Please ensure that you include a title page within your main document. We do appreciate that you have a title page document uploaded as a separate file, however, as per our author guidelines (http://journals.plos.org/plosone/s/submission-guidelines#loc-title-page) we do require this to be part of the manuscript file itself and not uploaded separately.

Could you therefore please include the title page into the beginning of your manuscript file itself, listing all authors and affiliations.

**Response:** The title page has been included within the main document.

3. We note that you have indicated that data from this study are available upon request. PLOS only allows data to be available upon request if there are legal or ethical restrictions on sharing data publicly. For information on unacceptable data access restrictions, please see http://journals.plos.org/plosone/s/data-availability#loc-unacceptable-data-access-restrictions.

We will update your Data Availability statement on your behalf to reflect the information you provide.

**Response:** The data availability is provided and attached.

In your revised cover letter, please address the following prompts:

4. Please include captions for your Supporting Information files at the end of your manuscript, and update any in-text citations to match accordingly. Please see our Supporting Information guidelines for more information: [http://journals.plos.org/plosone/s/supporting-information](about:blank).

**Response:** The caption for my Supporting Information file at the end of my manuscript is added.

**Comments to the Author**

1. Is the manuscript technically sound, and do the data support the conclusions?

The manuscript must describe a technically sound piece of scientific research with data that supports the conclusions. Experiments must have been conducted rigorously, with appropriate controls, replication, and sample sizes. The conclusions must be drawn appropriately based on the data presented.

Reviewer #1: Yes

Reviewer #2: Yes

**Response:** Thank you.

2. Has the statistical analysis been performed appropriately and rigorously?

Reviewer #1: Yes

Reviewer #2: Yes

**Response:** Thank you.

3. Have the authors made all data underlying the findings in their manuscript fully available?

The [PLOS Data policy](http://www.plosone.org/static/policies.action#sharing) requires authors to make all data underlying the findings described in their manuscript fully available without restriction, with rare exception (please refer to the Data Availability Statement in the manuscript PDF file). The data should be provided as part of the manuscript or its supporting information, or deposited to a public repository. For example, in addition to summary statistics, the data points behind means, medians and variance measures should be available. If there are restrictions on publicly sharing data—e.g. participant privacy or use of data from a third party—those must be specified.

Reviewer #1: Yes

Reviewer #2: No]

**Response:** The original data is in Persian which is translated and attached. .

4. Is the manuscript presented in an intelligible fashion and written in standard English?

PLOS ONE does not copyedit accepted manuscripts, so the language in submitted articles must be clear, correct, and unambiguous. Any typographical or grammatical errors should be corrected at revision, so please note any specific errors here.

Reviewer #1: Yes

Reviewer #2: Yes

**Response:** Thank you.

5. Review Comments to the Author

Please use the space provided to explain your answers to the questions above. You may also include additional comments for the author, including concerns about dual publication, research ethics, or publication ethics. (Please upload your review as an attachment if it exceeds 20,000 characters)

Reviewer #1: This manuscript entitled “Polyethylene eye cover versus artificial teardrops in the prevention of ocular surface diseases in comatose patients: a prospective multicenter randomized triple-blinded three-arm clinical trial” assess the effectivity of Polyethylene eye cover in comparison to other eye care treatments applied in UCI patients.

The sample included in each group is good, especially regarding the type of patients studied. In addition, the topic of the study is quite interesting since this population is normally invisible regarding eye care approach. Congrats to the authors for all their work!!

**Response:** Thank you.

In general, it is well explained and easy to read (very grateful for it!). I liked the introduction of the manuscript because it is a complete and clear introduction to the topic (it is a good point for someone who knows nothing about this matter). Also, the flow chart and tables are well executed, allowing a good interpretation of the data.

**Response:** Thank you.

I would like to give you some recommendation to improve your manuscript:

1. Be carefully with the acronyms. You should introduce them in the first mention and keep constant (it can be applied to all the manuscript). Please, check it (especially, in the introduction and discussion sections).

**Response:** Acronyms are checked and refined.

2. Please, check the English grammar and corrected (only a few words along the manuscript).

**Response:** Grammar is checked and refined.

3. The information about the ethical approval was duplicated (one in the ethical approval section and the other in the data collection section). Please, check it and eliminate one of them.

**Response:** It’s been eliminated.

4. I suggest creating a “statistical analysis” section and include both sample size information and statistical test performed in the study. In this way, it looks more organised.

**Response:** It’s been added.

5. I think that you should provide one or some references regarding your corneal staining grading. Is there any other study that performed in the same way you performed? Or, instead, if you decided to perform according to this method, why? Is it better than others reported in the literature? Please, add some information.

**Response:** They have been added and color coded in red.

I have missed more information about the evaluation of the staining (Did you leave the eyes open or close during this time? It is supposed that the patient cannot blink…therefore…how it takes into account? When you perform the measurements in those patients? During those 15 minutes of corneal oxygenation?).

**Response:** As we have mentioned, all interventions were provided over a 5-day period and the OSD incidence and severity were measured at baseline and post-intervention. Therefore, the eye examinations did not overlap with the interventions.

6. In methods, it is important to provide the manufactures (small detail) of the product/treatments that you have used. For example, authors have mentioned the use of fluorescein…you should add information about the volume/type of fluorescein (Did you use a strip with saline solution? or instead, a specific volume of fluorescein?). You should think that maybe other researchers want to reproduce your protocol…so, you must provide all the details. Please, applied to all the instruments or devices you used during your research.

**Response:** They have been added and color coded in red.

7. Regarding the discussion, I think it is correct (also in length) but I think could be improved. I suggest you start talking about your findings, follow by the comparison between treatments (previous studies), follow by the relevance of Polyethylene eye cover and finally your conclusions. I think you should highlight better the findings of your study (it does not seem as much supported by evidence than your introduction).

**Response:** It has been modified and colored in red.

Additionally, I would like to express some impressions and comments about the manuscript.

1. Despite the great work that you have done…I’m little bit disappointed regarding ocular examination. I think that the evaluation could be more complete than only the corneal damage assessment. Despite keratopathy is the principal ocular complication, both chemosis and microbial keratitis are important in this population.
In fact, this method is particularly useful in patients with chemosis and lagopthalmos (according to the literature, especially including mechanical ventilated patients). Therefore, I think that the assessment of conjunctival integrity (staining and hyperaemia), as well as tear volume, could add valuable information. Previous studies have also included conjunctiva cultures which is a good approach to confirm the microbial presence (very important in this population due to the time that must spent in this condition). I suggest you include a more clinical and diagnostic test in your next study to provide a wider evaluation of the ocular surface.

**Response:** Yes, you are right. Further studies are required to consider the chemosis, exudate, tear volume, dry eyes, and microbial keratitis as the eye-care intervention outcomes.

2. Regarding the treatment that you choose to compare…I have some doubts. I understand that you include normal saline because it is included in the routine care in Iran and it is your “gold standard”. Despite this, it is well-known (as you mention as well in your introduction and discussion sections) that normal saline is no well supported because of its consequences in the ocular surface. For this reason, it is more than evident that compare this treatment with the others…it will show worst results. In addition to it, it has been proved in the literature (that artificial tears are better in comparison to normal saline and even Polyethylene eye cover versus artificial tears). Therefore, I think the results are quite expected. I think that it would be more interesting to compare two or three treatments that previously have shown a similar behaviour in this population and evaluate which one it is better.

**Response:** Unfortunately, not only in Iran but in many countries the NS irrigation is common and sometimes is the only routine eye-care. This study reconfirmed the effectiveness of the polyethylene eye-cover and explained how to cover the eyes in detail. More studies are needed for determining the schedule of exchanging eye covers in order to maintain the eyes’ surface oxygenation.

Reviewer #2: The authors consider an interesting design in this manuscript to assess the efficacy of polyethylene eye cover in preventing occular surface diseases. The statistical study design (2-staged randomization) considered, and the analysis plan, although looking OK, require further clarification, along the lines stated below:

**Response:** Thank you.

(1) The study is multicenter, however, that structure is not considered in the calculation of the statistical power, or the analysis aspects. To be specific, more argument is necessary on the iid (independent and identically distributed) assumptions of data generated from the 3 ICUs. I mean, can they be considered homogenous? If not, one may need to resort to application of more complex tests, like the clustered McNemar test (see reference a below), or the clustered nonparametric rank sum/Kruskal-Wallis tests (see reference b).

References:
(a) https://www.ncbi.nlm.nih.gov/pubmed/15236431
(b) https://www.tandfonline.com/doi/abs/10.1198/016214504000001583

**Response:** You are right. The ICU patients are complex and different even in the same ICU, and that is why we considered only one eye of every patient to each study arm. In this way we tried to control the possible differences among participants such as clinical and settings conditions that may influence the eyes’ status.

As shown in table 3, we have compared every single eye with each other, not patients. .

(2) Provide the full name of K-S (I assume Kolmogorov-Smirnov) test when introduced for the first time, in Page 8

**Response:** replaced.

(3) The sample size calculation uses a proportion test; for more clarity, provide justification of using such a test, including the effect-size considered. The outcome you are considering (such as the OSD severity, etc) are not really proportion responses, right? So, is this calculation appropriate, or you need to use the correct test for calculations?

**Response:** We set the sample size calculation on the incidence (proportion) of OSD in comatose patients. We also explain the post hoc effect size for the power of test in the text and colored in red, as follow:

In a post hoc power analysis by G*Power 3.1.9.2 software, when we set a small effect size 0.25, a significance level of α = .05, with total sample size 158 in three groups, the achieved power computed more than 0.80.

Also, the current version is full of typos, and misconstructs; those need to be corrected. Some are:
. confidential level should be confidence level
. Page 2, Introduction, 3rd sentence: Remove "to"
. Page 5, penultimate paragraph, change "applicable" to "apply".

There are several others, and I leave it upon the authors to rectify.

**Response:** They are corrected.
