# Supplementary material for: Polyethylene eye-cover versus artificial teardrops in the prevention of ocular surface diseases in comatose patients: A prospective multicenter randomized triple-blinded three-arm clinical trial
Source: PLoS One. 2021 Apr 1;16(4):e0248830. doi: 10.1371/journal.pone.0248830 (PMC8016328; doi:10.1371/journal.pone.0248830)
Supplement: Attachment — Submitted filename: Second-Responses to the reviewers .docx [file pone.0248830.s017.docx]

**Dear Dr. Joseph Donlan,**

Thank you for your email and for the reviewers' comments concerning our manuscript entitled  ”Polyethylene eye cover versus artificial teardrops in the prevention of ocular surface diseases in comatose patients: a prospective multicenter randomized triple-blinded three-arm clinical trial” no: PONE-D-19-33881R1.

We have studied comments carefully and have made revised which we hope meet with approval. Revised portion are highlighted in the manuscript. The main corrections in the paper and the responds to the Editor’s and reviewer’s comments are as following:

| Questions | Reviewer #1 | Reviewer #2 | Reviewer #3 | Reviewer #4 | Response |
| --- | --- | --- | --- | --- | --- |
| 1. If the authors have adequately addressed your comments raised in a previous round of review. | All comments have been addressed | All comments have been addressed | All comments have been addressed | All comments have been addressed | Thank you. |
| 2. Is the manuscript technically sound, and do the data support the conclusions? | Yes | - | Partly | Partly | The original data was modified in English and attached. |
| 3. Has the statistical analysis been performed appropriately and rigorously? | Yes | Yes | Yes | Yes | Thank you. |
| 4. Have the authors made all data underlying the findings in their manuscript fully available? | Yes | Yes | - | Yes | Thank you. |
| 5. Is the manuscript presented in an intelligible fashion and written in standard English? | Yes | - | Yes | Yes | Thank you. |

**6. Review Comments to the Author**

**Reviewer #1:**

- Thank you to authors to take into account all the suggestions made.

Response: Thank you too.

- Despite most of the comments have been addressed, several grammar mistakes, repeated words or wrong spelling still remain in the manuscript. Here I detail some of them:

Line 9 (INTRODUCTION): Conjunctival infections to. serious everlasting...

Response: Corrected.

Line 22 (INTRODUCTION): Applying polyethylene eye covers has (BEEN) suggested as the most...

Response: Corrected.

Line 26 (INTRODUCTION): So that, the standard care has been only COMPRISED COMPROMISED a daily washing...

Response: Corrected.

Fig 1: Fallow-up (Follow-up)

Discussion: Our findings are also...by Sahn and Min in showing...as found by Shan and Min (redundant due to repetition)

Response: Corrected.

**Reviewer #2: Nothing**

Response: -

**Reviewer #3:**

- The authors have compared using normal saline eye drop versus lubricant eye drops versus polyethylene eye covers for study of ocular surface disease in comatose patients. Though it is an important topic to be addressed, there are some basic issues with the manuscript. The results are expected, and add limited knowledge to existing literature.

Response: As far as the authors know, this is the first prospective multicenter randomized triple-blinded three-arm clinical trial in which each patient was a member of the control and experimental group simultaneously. Furthermore, we considered changing the eye-covers every 12 hours with a 15 minutes lag for corneal oxygenation, not like the other studies which had a 24-hour schedule without considering the natural cornea oxygenation. We studied both the incidence and severity of OSD in ICU patients. However, there are many spaces for such a study, and we think that explaining the methodology in detail can help and facilitate future studies for other eye care methods in ICUs.

- 1. Page 4, line 5: “So, that,,,” This line appears to be misquoted. The standard care for authors of reference 20 was that. It cannot be considered as the universal standard of care. Standards should be quoted based on guidelines – for example, The Royal college guidelines: https://www.rcophth.ac.uk/wp-content/uploads/2017/11/Intensive-Care-Unit.pdf. In fact, these guidelines are against the usage of NS, and suggest warm water bath.

Response: Corrected.

- 2. Page 4, paragraph 2, line 1: “In Iran…” This line and the authors’ reply to reviewer 1 states that NS washing is routine eye care in ICU. Below is a paper from Iran which also proposes against the use of NS.

Impact of Normal Saline on the Incidence of Exposure Keratopathy in Patients Hospitalized in Intensive Care Units. Iran J Nurs Midwifery Res. 2018 Jan-Feb; 23(1): 57–60. doi: 10.4103/ijnmr.IJNMR_187_16

It is ironic why it is still continued as a routine care in Iran, despite being proven adverse. The authors also acknowledge this in their critical appraisal, yet have continued to do so, which is disappointing.

Response: NS is not recommended anymore, yet unfortunately, it is routine care in the ICUs in Iran and many in developing countries. As we mentioned in the text, we added NS drops, teardrops, and eye covering to the routine care (irrigation the eyes with NS). So, the first step in our three ways of interventions was very gentle lids and skin washing by NS. Your comments will be a good idea for future clinical trials about using sterile water instead of NS to clean the lids and crusts.

- 3. Was it the same blinded examiner who studied the patients in all the 3 ICUs?

Response: Yes. It was written as below in text: A single observer who was blinded as to the patient assignments evaluated the patients’ eyes based on the Corneal Fluorescein Staining Pattern.

- 4. Including all comatose patients in this study could have led to bias. For example, a cornea exposed eye being administered just 2 drops of NS Q6H, is likely to develop OSD while only conjunctiva exposed eye or a closed eye may not.

As per standard guidelines (Ref: https://www.rcophth.ac.uk/wp-content/uploads/2017/11/Intensive-Care-Unit.pdf), eyes which have grade 0 closure need no action at all, and eyes with only conjunctival exposure (grade-1) will have unaffected corneas even otherwise. Since the authors are only looking for corneal manifestations, ideally only grade-2 exposed eyes should have been included.

Response: As we mentioned, an ophthalmologist confirmed whether the patients’ eyes had the study inclusion criteria or not. Comparing was done between two eyes of the same patient who was simultaneously under 2 methods, so, the exposure of both eyes was the same.

- 5. It is said that all eyes had a healthy cornea (confirmed by ophthalmologist) at the start of the study. After institution of therapy, when were they re-examined – at the end of 5 day period? Was the examination time kept constant for all eyes, like for example, 1 hour after last therapy?

Response: Explained.

- 6. Elaborating the results on how many eyes had +1 grading due to the mentioned criteria, and their breakdown could be informative.

Response: Please find Table 3 where we wrote the number of the eyes with different severity grades of OSD criteria in each group separately.

- 7. A photograph of the polyethylene film applied can make the manuscript more appealing.

Response: The procedure of eye covering was according to what was advised in the references bellow:

Sharjeel M, Malik IQ, Iqbal CJ, Ali F. Prevention of exposure keratopathy with sahaf wet chamber. Pakistan Journal of Ophthalmology. 2015;31(3):131-6.

Al-Ribh SA, Baker RH, Gupta RK, Al Dossary TS. Polyethylene cover versus viscotears gel for the prevention of corneal abrasions in critically ill patients: a comparative study. Journal of King Abdulaziz University-Medical Sciences. 2012;19(1 Sup):59-72. doi: https://doi.org/10.4197/Med.19-1S.4.

- 8. As per reviewer 1’s comments and my concern, it is important to add on to limitations about missing on the other forms of OSD in unconscious patients such as epithelial defects, infections, tear studies and conjunctival manifestations.

Response: Added.

- 9. References 17 & 19 are the same. Please remove one.

Response: Corrected.

- 10. Were they tear drops or ointments – please provide the type and concentration of the lubricant along with manufacturer details.

Response: Added.

- 11. Supplementary detailed results, table 7, 8: Please either give row % or column %. The combination of both appears confusing.	.

Response: Corrected.

- 12. Supplementary detailed results, table 9: The data between table 9 and table 7,8 does not tally for artificial tear drops and polyethylene cover. For example, table 8 shows a total of 8 OSD eyes, but table 9 shows only 7 OSD cases.

Response: Corrected.

- 13. For table 3 and supplementary table 9 and 15: Please give the individual p values between the groups.

Response: Tables of comparing of the incidence and severity between two groups are added in the supplementary.

- 14. In my opinion, the discussion needs to stress against the use of NS drops/irrigation so that the practice in Iran could change.

Response: Added.

- Minor comments: Introduction line 4: but ‘receive’ minimal.

Response: Corrected.

- Introduction line 7: Do the authors mean ‘altered’ tear components concentration?

Response: Corrected.

- Page 3, paragraph 2, line 4: I think washing eyes with NS should come last, as it is proven to show adverse effects.

Response: NS irrigation was introduced by many references we used for that paragraph (References 3, 4, 8, 10, 12-17). So, may be better to come first.

- Figure 1: correct ‘follow-up’

Response: Corrected.

- Figure 1: ‘lost to follow-up due to meet exclusion criteria’ – rephrase/explain

Response: Explained.

- Table 1: Does Internal patients mean internal medicine department patients? Please clarify internal patients.

Response: Explained.

- Page 13, line 4 appears out of context. May be more suitable in the subsequent paragraph.

Response: Corrected.

- Page 13, line 14: Pls mention what was the other intervention

Response: Explained.

- Page 13, line 18: Recheck the sentence

Response: Corrected.

- Page 13, last paragraph first line: This line is misleading. Reference 4 clearly states against cotton wool balls and NS irrigation in its ‘DO-NOTs’ section.

Response: Corrected.

- Page 14, line 7: ‘pre-disposable’ polyethylene eye cover – meaning?

Response: We cut pieces of 10*10 polyethylene film (clear plastic film) by scissors for every patient and fixed them over the patients’ eyes with adhesive tapes. If these plastic pieces with the edge adhesive ability were ready, the time and using them would have been lesser and easier.

- Supporting information file: ‘original’ results

Response: Added.

**Reviewer #4:**

- However, in order to confer greater scientific criticality and greater intelligibility of the text of the article, I describe some considerations below:
- 1.Title: Clear, attractive and relevant to the text.

Response: Thank you.

- 2.Abstract: Well structured and coherent.

Response: Thank you.

- 3.Keywords: I suggest modifying “Infectious Diseases” by “Eye Diseases”, as diseases of the ocular surface are not limited to infectious diseases.

Response: Modified.

- 4. Introduction
- Although well described, targeted and synthetic, I missed a definition of ocular surface diseases in the introduction and possible diagnostic methods.

Response: Added.

- In addition, it needs to describe more clearly what the current study shows different/new when compared to other clinical trials that also compared polyethylene covers with artificial tears.

As far as the authors know,

1- This is the first prospective multicenter randomized triple-blinded three-arm clinical trial in this regards.
2- During the study, we considered changing the eye-covers every 12 hours with a 15 minutes lag for corneal oxygenation. Other studies had a 24-hour schedule without considering the cornea oxygenation.
3- In our study, each patient was a member of the control and/or experimental group simultaneously.
4- We studied both the incidence and severity of OSD in ICU patients in which the results showed both incidence and severity of OSD depended on the eye care method.

However, there are many spaces for such a study, and we think that explaining the methodology in detail can help and facilitate future studies for other eye care methods in ICUs.

- 5. Materials and methods
- Describe more clearly how ocular surface disease was established/diagnosed and what degrees of fluorescein testing were considered to classify OSD?

Response: Explained.

- Justify the reason for having followed up for a period of five days. In addition, it is not clear whether all patients were followed up entirely during the five days or whether they were followed up until the appearance of changes in the fluorescein test indicating the presence of the outcome (OSD).

Response: Added.

- It was not clear at what time the cornea was evaluated with fluorescein staining. Was it used daily? At the same times for all patients? What procedures were performed before the application of fluorescein that allowed the blinding of the ophthalmologist?

Response: Explained.

- In data collection, when the researchers describe the data on the demographic and clinical characteristics of the patients (Age, sex and type of injury), in fact, what appears in the results is not the type of injury, but the reason for ICU admission. Then, I suggest adjusting this information.

Response: Corrected.

- I suggest describing manufacturers/ specific brands of interventions/ treatments (Polyethylene cover, artificial teardrops and normal saline), as well as briefly describing the presentation characteristics of each one.

Response: In our home university, we are inhibited to name the brand of materials because of advertisement and potential conflict of interest. However, more details of these materials are added.

- 6. Results
- According to CONSORT, the flow diagram must be presented in the results.

Response: Replaced.

- What does “Internal patients” mean for reasons of hospitalization?

Response: Explained.

- The information " The OSDs were observed in 66 (41.77%) out of 158 eyes examined" appears only in the abstract of the work. Therefore, it must also be included in the search results.

Response: Added.

- In the results, the authors demonstrated the presence or not of statistical association between comparisons of interventions. However, they did not indicate/describe the magnitude of the association, using, for example, the Relative Risk with their respective confidence intervals.

Response: Tables of comparing of the incidence and severity between two groups are added in the supplementary.

- 7. Discussion
- It provides a logical and appropriate interpretation of results in an expanded scientific context and manages to engage in a targeted dialogue with literature and other studies.

Response: Thank you.

- 8. Conclusion
- Precise, clear and consistent with the purpose of the study.

Response: Thank you.

- 9. References
- Relevant and appropriate to the text and theme.

Response: Thank you.

**Best regards,**

**Dr. Mahnaz Khatiban**
