# Supplementary material for: Polyethylene eye-cover versus artificial teardrops in the prevention of ocular surface diseases in comatose patients: A prospective multicenter randomized triple-blinded three-arm clinical trial
Source: PLoS One. 2021 Apr 1;16(4):e0248830. doi: 10.1371/journal.pone.0248830 (PMC8016328; doi:10.1371/journal.pone.0248830)
Supplement: Attachment — Submitted filename: 4th Responses to the reviewers .docx [file pone.0248830.s018.docx]

**Dear Dr. Emma Darkin,**

Thank you for your email and for the reviewers' comments concerning our manuscript entitled  ”Polyethylene eye cover versus artificial teardrops in the prevention of ocular surface diseases in comatose patients: a prospective multicenter randomized triple-blinded three-arm clinical trial” no: PONE-D-19-33881R1.

We have studied the last comments carefully and have made minor revision as you wish.

Revised portion are highlighted in the manuscript. The main corrections in the paper and the responds to the Editor’s and reviewer’s comments are as following:

6. Review Comments to the Author

**Reviewer #1:** The authors have addressed all of my recommendations. Together with the rest of the reviewers' recommendations, the manuscript has been improved. As a final comment, I suggest the authors check the acronyms again. Be sure that all of them were defined previously.

**Response**: Thank you very much. The acronyms are checked and refined.

**Reviewer #2:** (No Response)

**Response**: -

**Reviewer #3:** • An English revision is required.

**Response**: The English editing is checked and refined.

• I doubt if my previous comment #4 was misunderstood: “Including all comatose patients in this study could have led to bias. For example, a cornea exposed eye being administered just 2 drops of NS Q6H, is likely to develop OSD while only conjunctiva exposed eye or a closed eye may not. As per standard guidelines (Ref: https://www.rcophth.ac.uk/wpcontent/uploads/2017/11/Intensive-Care-Unit.pdf), eyes which have grade 0 closure need no action at all, and eyes with only conjunctival exposure (grade-1) will have unaffected corneas even otherwise. Since the authors are only looking for corneal manifestations, ideally only grade-2 exposed eyes should have been included”. I meant that including only cornea exposed eyes would have given more specific results, and did not mean the inter-eye symmetry. Or, the exposure should have been graded 0-2, and each grade analyzed individually. The inclusion criteria the authors have stated appear to be primarily related to their inability to blink and a normal cornea at the start.

However, this is a bias related to the core of the study, and I would suggest the authors to add this point under the limitations.

**Response**: Although our eligibility criteria were included incomplete eyelids closure too, the grades of eye closure were not our main criterion. So, this point is added to the limitations.

• Previous comment #6 was questioned about “A score of severity is added when the PEE was seen in the central 4mm diameter portion of the cornea, one or more filaments occurred anywhere on the cornea, or one or more patches of confluent staining, including linear stains, are found anywhere on the cornea”

**Response**: More detail is explained.

• Previous last minor comment was to replace ‘origin’ results by ‘original’ results at the legend for supporting file. I’m sorry that it was misunderstood. The present supplementary tables are too numerous and confusing, please keep the previous tables itself, but correct the numerical errors as explained previously.

**Response**: Replaced.

*****************

**Best regards,**

**Dr. Mahnaz Khatiban**
