# Supplementary material for: Polyethylene eye-cover versus artificial teardrops in the prevention of ocular surface diseases in comatose patients: A prospective multicenter randomized triple-blinded three-arm clinical trial
Source: PLoS One. 2021 Apr 1;16(4):e0248830. doi: 10.1371/journal.pone.0248830 (PMC8016328; doi:10.1371/journal.pone.0248830)
Supplement: Peer review history

# Peer review: Decision Letter 0

**DOI:** 10.1371/journal.pone.0248830.r001

**Authors:** José M. González-Méijome

29 Apr 2020

PONE-D-19-33881

Polyethylene eye cover versus artificial teardrops in the prevention of ocular surface diseases in comatose patients: a prospective multicenter randomized triple-blinded three-arm clinical trial

PLOS ONE

Dear Mr. Falahinia,

Thank you for submitting your manuscript to PLOS ONE. After careful consideration, we feel that it has merit but does not fully meet PLOS ONE’s publication criteria as it currently stands. Therefore, we invite you to submit a revised version of the manuscript that addresses the points raised during the review process.

Both reviewers raised significant issues in your manuscript. This includes several statistical and methodological aspects that deserve further clarification. 

We would appreciate receiving your revised manuscript by Jun 13 2020 11:59PM. When you are ready to submit your revision, log on to https://www.editorialmanager.com/pone/ and select the 'Submissions Needing Revision' folder to locate your manuscript file.

If you would like to make changes to your financial disclosure, please include your updated statement in your cover letter.

To enhance the reproducibility of your results, we recommend that if applicable you deposit your laboratory protocols in protocols.io, where a protocol can be assigned its own identifier (DOI) such that it can be cited independently in the future. For instructions see: http://journals.plos.org/plosone/s/submission-guidelines#loc-laboratory-protocols

Please include the following items when submitting your revised manuscript:

- A rebuttal letter that responds to each point raised by the academic editor and reviewer(s). This letter should be uploaded as separate file and labeled 'Response to Reviewers'.
- A marked-up copy of your manuscript that highlights changes made to the original version. This file should be uploaded as separate file and labeled 'Revised Manuscript with Track Changes'.
- An unmarked version of your revised paper without tracked changes. This file should be uploaded as separate file and labeled 'Manuscript'.

Please note while forming your response, if your article is accepted, you may have the opportunity to make the peer review history publicly available. The record will include editor decision letters (with reviews) and your responses to reviewer comments. If eligible, we will contact you to opt in or out.

We look forward to receiving your revised manuscript.

Kind regards,

José M. González-Méijome, PhD

Academic Editor

PLOS ONE

Journal Requirements:

When submitting your revision, we need you to address these additional requirements.

1. Please ensure that your manuscript meets PLOS ONE's style requirements, including those for file naming. The PLOS ONE style templates can be found at https://journals.plos.org/plosone/s/file?id=wjVg/PLOSOne_formatting_sample_main_body.pdf and https://journals.plos.org/plosone/s/file?id=ba62/PLOSOne_formatting_sample_title_authors_affiliations.pdf

2. Please ensure that you include a title page within your main document. We do appreciate that you have a title page document uploaded as a separate file, however, as per our author guidelines (http://journals.plos.org/plosone/s/submission-guidelines#loc-title-page) we do require this to be part of the manuscript file itself and not uploaded separately.

Could you therefore please include the title page into the beginning of your manuscript file itself, listing all authors and affiliations.

3. We note that you have indicated that data from this study are available upon request. PLOS only allows data to be available upon request if there are legal or ethical restrictions on sharing data publicly. For information on unacceptable data access restrictions, please see http://journals.plos.org/plosone/s/data-availability#loc-unacceptable-data-access-restrictions.

In your revised cover letter, please address the following prompts:

a) If there are ethical or legal restrictions on sharing a de-identified data set, please explain them in detail (e.g., data contain potentially identifying or sensitive patient information) and who has imposed them (e.g., an ethics committee). Please also provide contact information for a data access committee, ethics committee, or other institutional body to which data requests may be sent.

b) If there are no restrictions, please upload the minimal anonymized data set necessary to replicate your study findings as either Supporting Information files or to a stable, public repository and provide us with the relevant URLs, DOIs, or accession numbers. Please see http://www.bmj.com/content/340/bmj.c181.long for guidelines on how to de-identify and prepare clinical data for publication. For a list of acceptable repositories, please see http://journals.plos.org/plosone/s/data-availability#loc-recommended-repositories.

We will update your Data Availability statement on your behalf to reflect the information you provide.

4. Please include captions for your Supporting Information files at the end of your manuscript, and update any in-text citations to match accordingly. Please see our Supporting Information guidelines for more information: http://journals.plos.org/plosone/s/supporting-information.

Additional Editor Comments (if provided):

[Note: HTML markup is below. Please do not edit.]

Reviewers' comments:

Reviewer's Responses to Questions

**Comments to the Author**

1. Is the manuscript technically sound, and do the data support the conclusions?

The manuscript must describe a technically sound piece of scientific research with data that supports the conclusions. Experiments must have been conducted rigorously, with appropriate controls, replication, and sample sizes. The conclusions must be drawn appropriately based on the data presented. 

Reviewer #1: Yes

Reviewer #2: Yes

**********

2. Has the statistical analysis been performed appropriately and rigorously? 

Reviewer #1: Yes

Reviewer #2: Yes

**********

3. Have the authors made all data underlying the findings in their manuscript fully available?

The PLOS Data policy requires authors to make all data underlying the findings described in their manuscript fully available without restriction, with rare exception (please refer to the Data Availability Statement in the manuscript PDF file). The data should be provided as part of the manuscript or its supporting information, or deposited to a public repository. For example, in addition to summary statistics, the data points behind means, medians and variance measures should be available. If there are restrictions on publicly sharing data—e.g. participant privacy or use of data from a third party—those must be specified.

Reviewer #1: Yes

Reviewer #2: No

**********

4. Is the manuscript presented in an intelligible fashion and written in standard English?

PLOS ONE does not copyedit accepted manuscripts, so the language in submitted articles must be clear, correct, and unambiguous. Any typographical or grammatical errors should be corrected at revision, so please note any specific errors here.

Reviewer #1: Yes

Reviewer #2: Yes

**********

5. Review Comments to the Author

Please use the space provided to explain your answers to the questions above. You may also include additional comments for the author, including concerns about dual publication, research ethics, or publication ethics. (Please upload your review as an attachment if it exceeds 20,000 characters)

Reviewer #1: This manuscript entitled “Polyethylene eye cover versus artificial teardrops in the prevention of ocular surface diseases in comatose patients: a prospective multicenter randomized triple-blinded three-arm clinical trial” assess the effectivity of Polyethylene eye cover in comparison to other eye care treatments applied in UCI patients.

The sample included in each group is good, especially regarding the type of patients studied. In addition, the topic of the study is quite interesting since this population is normally invisible regarding eye care approach. Congrats to the authors for all their work!!

In general, it is well explained and easy to read (very grateful for it!). I liked the introduction of the manuscript because it is a complete and clear introduction to the topic (it is a good point for someone who knows nothing about this matter). Also, the flow chart and tables are well executed, allowing a good interpretation of the data.

I would like to give you some recommendation to improve your manuscript:

1. Be carefully with the acronyms. You should introduce them in the first mention and keep constant (it can be applied to all the manuscript). Please, check it (especially, in the introduction and discussion sections)

2. Please, check the English grammar and corrected (only a few words along the manuscript).

3. The information about the ethical approval was duplicated (one in the ethical approval section and the other in the data collection section). Please, check it and eliminate one of them.

4. I suggest creating a “statistical analysis” section and include both sample size information and statistical test performed in the study. In this way, it looks more organised.

5. I think that you should provide one or some references regarding your corneal staining grading. Is there any other study that performed in the same way you performed? Or, instead, if you decided to perform according to this method, why? Is it better than others reported in the literature? Please, add some information.

I have missed more information about the evaluation of the staining (Did you leave the eyes open or close during this time? It is supposed that the patient cannot blink…therefore…how it takes into account? When you perform the measurements in those patients? During those 15 minutes of corneal oxygenation?).

6. In methods, it is important to provide the manufactures (small detail) of the product/treatments that you have used. For example, authors have mentioned the use of fluorescein…you should add information about the volume/type of fluorescein (Did you use a strip with saline solution? or instead, a specific volume of fluorescein?). You should think that maybe other researchers want to reproduce your protocol…so, you must provide all the details. Please, applied to all the instruments or devices you used during your research.

7. Regarding the discussion, I think it is correct (also in length) but I think could be improved. I suggest you start talking about your findings, follow by the comparison between treatments (previous studies), follow by the relevance of Polyethylene eye cover and finally your conclusions. I think you should highlight better the findings of your study (it does not seem as much supported by evidence than your introduction).

Additionally, I would like to express some impressions and comments about the manuscript.

1. Despite the great work that you have done…I’m little bit disappointed regarding ocular examination. I think that the evaluation could be more complete than only the corneal damage assessment. Despite keratopathy is the principal ocular complication, both chemosis and microbial keratitis are important in this population.

In fact, this method is particularly useful in patients with chemosis and lagopthalmos (according to the literature, especially including mechanical ventilated patients). Therefore, I think that the assessment of conjunctival integrity (staining and hyperaemia), as well as tear volume, could add valuable information. Previous studies have also included conjunctiva cultures which is a good approach to confirm the microbial presence (very important in this population due to the time that must spent in this condition). I suggest you include a more clinical and diagnostic test in your next study to provide a wider evaluation of the ocular surface.

2. Regarding the treatment that you choose to compare…I have some doubts. I understand that you include normal saline because it is included in the routine care in Iran and it is your “gold standard”. Despite this, it is well-known (as you mention as well in your introduction and discussion sections) that normal saline is no well supported because of its consequences in the ocular surface. For this reason, it is more than evident that compare this treatment with the others…it will show worst results. In addition to it, it has been proved in the literature (that artificial tears are better in comparison to normal saline and even Polyethylene eye cover versus artificial tears). Therefore, I think the results are quite expected. I think that it would be more interesting to compare two or three treatments that previously have shown a similar behaviour in this population and evaluate which one it is better.

Reviewer #2: The authors consider an interesting design in this manuscript to assess the efficacy of polyethylene eye cover in preventing occular surface diseases. The statistical study design (2-staged randomization) considered, and the analysis plan, although looking OK, require further clarification, along the lines stated below:

(1) The study is multicenter, however, that structure is not considered in the calculation of the statistical power, or the analysis aspects. To be specific, more argument is necessary on the iid (independent and identically distributed) assumptions of data generated from the 3 ICUs. I mean, can they be considered homogenous? If not, one may need to resort to application of more complex tests, like the clustered McNemar test (see reference a below), or the clustered nonparametric rank sum/Kruskal-Wallis tests (see reference b).

(2) Provide the full name of K-S (I assume Kolmogorov-Smirnov) test when introduced for the first time, in Page 8

(3) The sample size calculation uses a proportion test; for more clarity, provide justification of using such a test, including the effect-size considered. The outcome you are considering (such as the OSD severity, etc) are not really proportion responses, right? So, is this calculation appropriate, or you need to use the correct test for calculations?

Also, the current version is full of typos, and misconstructs; those need to be corrected. Some are:

. confidential level should be confidence level

. Page 2, Introduction, 3rd sentence: Remove "to"

. Page 5, penultimate paragraph, change "applicable" to "apply".

There are several others, and I leave it upon the authors to rectify.

References:

(a) https://www.ncbi.nlm.nih.gov/pubmed/15236431

(b) https://www.tandfonline.com/doi/abs/10.1198/016214504000001583

**********

6. PLOS authors have the option to publish the peer review history of their article (what does this mean?). If published, this will include your full peer review and any attached files.

If you choose “no”, your identity will remain anonymous but your review may still be made public.

**Do you want your identity to be public for this peer review?** For information about this choice, including consent withdrawal, please see our Privacy Policy.

Reviewer #1: No

Reviewer #2: No

[NOTE: If reviewer comments were submitted as an attachment file, they will be attached to this email and accessible via the submission site. Please log into your account, locate the manuscript record, and check for the action link "View Attachments". If this link does not appear, there are no attachment files to be viewed.]

While revising your submission, please upload your figure files to the Preflight Analysis and Conversion Engine (PACE) digital diagnostic tool, https://pacev2.apexcovantage.com/. PACE helps ensure that figures meet PLOS requirements. To use PACE, you must first register as a user. Registration is free. Then, login and navigate to the UPLOAD tab, where you will find detailed instructions on how to use the tool. If you encounter any issues or have any questions when using PACE, please email us at figures@plos.org. Please note that Supporting Information files do not need this step.

# Author comment: Author response to Decision Letter 0

**DOI:** 10.1371/journal.pone.0248830.r002

25 Jun 2020

Dear Editors and Reviewers,

Thank you for your email and for the reviewers' comments concerning our manuscript entitled  ”Polyethylene eye cover versus artificial teardrops in the prevention of ocular surface diseases in comatose patients: a prospective multicenter randomized triple-blinded three-arm clinical trial” no: PONE-D-19-33881. Those comments are all valuable and very helpful for re-revising and improving our manuscript, as well as the important guiding significance to our researches. We have studied comments carefully and have made revised which we hope meet with approval. Revised portion are highlighted in the manuscript. The main corrections in the paper and the responds to the Editor’s and reviewer’s comments are as flowing: 

Journal Requirements:

When submitting your revision, we need you to address these additional requirements.

1. Please ensure that your manuscript meets PLOS ONE's style requirements, including those for file naming. The PLOS ONE style templates can be found at https://journals.plos.org/plosone/s/file?id=wjVg/PLOSOne_formatting_sample_main_body.pdf and https://journals.plos.org/plosone/s/file?id=ba62/PLOSOne_formatting_sample_title_authors_affiliations.pdf

Response: My manuscript comes across PLOS ONE's style requirements.

2. Please ensure that you include a title page within your main document. We do appreciate that you have a title page document uploaded as a separate file, however, as per our author guidelines (http://journals.plos.org/plosone/s/submission-guidelines#loc-title-page) we do require this to be part of the manuscript file itself and not uploaded separately.

Could you therefore please include the title page into the beginning of your manuscript file itself, listing all authors and affiliations.

Response: The title page has been included within the main document.

3. We note that you have indicated that data from this study are available upon request. PLOS only allows data to be available upon request if there are legal or ethical restrictions on sharing data publicly. For information on unacceptable data access restrictions, please see http://journals.plos.org/plosone/s/data-availability#loc-unacceptable-data-access-restrictions.

We will update your Data Availability statement on your behalf to reflect the information you provide.

Response: The data availability is provided and attached.

In your revised cover letter, please address the following prompts:

4. Please include captions for your Supporting Information files at the end of your manuscript, and update any in-text citations to match accordingly. Please see our Supporting Information guidelines for more information: http://journals.plos.org/plosone/s/supporting-information.

Response: The caption for my Supporting Information file at the end of my manuscript is added.

Comments to the Author

1. Is the manuscript technically sound, and do the data support the conclusions?

The manuscript must describe a technically sound piece of scientific research with data that supports the conclusions. Experiments must have been conducted rigorously, with appropriate controls, replication, and sample sizes. The conclusions must be drawn appropriately based on the data presented.

Reviewer #1: Yes

Reviewer #2: Yes

Response: Thank you.

________________________________________

2. Has the statistical analysis been performed appropriately and rigorously?

Reviewer #1: Yes

Reviewer #2: Yes

 Response: Thank you.

________________________________________

3. Have the authors made all data underlying the findings in their manuscript fully available?

The PLOS Data policy requires authors to make all data underlying the findings described in their manuscript fully available without restriction, with rare exception (please refer to the Data Availability Statement in the manuscript PDF file). The data should be provided as part of the manuscript or its supporting information, or deposited to a public repository. For example, in addition to summary statistics, the data points behind means, medians and variance measures should be available. If there are restrictions on publicly sharing data—e.g. participant privacy or use of data from a third party—those must be specified.

Reviewer #1: Yes

Reviewer #2: No]

 Response: The original data is in Persian which is translated and attached. .

________________________________________

4. Is the manuscript presented in an intelligible fashion and written in standard English?

PLOS ONE does not copyedit accepted manuscripts, so the language in submitted articles must be clear, correct, and unambiguous. Any typographical or grammatical errors should be corrected at revision, so please note any specific errors here.

Reviewer #1: Yes

Reviewer #2: Yes

 Response: Thank you.

________________________________________

5. Review Comments to the Author

Please use the space provided to explain your answers to the questions above. You may also include additional comments for the author, including concerns about dual publication, research ethics, or publication ethics. (Please upload your review as an attachment if it exceeds 20,000 characters)

Reviewer #1: This manuscript entitled “Polyethylene eye cover versus artificial teardrops in the prevention of ocular surface diseases in comatose patients: a prospective multicenter randomized triple-blinded three-arm clinical trial” assess the effectivity of Polyethylene eye cover in comparison to other eye care treatments applied in UCI patients.

The sample included in each group is good, especially regarding the type of patients studied. In addition, the topic of the study is quite interesting since this population is normally invisible regarding eye care approach. Congrats to the authors for all their work!!

Response: Thank you.

In general, it is well explained and easy to read (very grateful for it!). I liked the introduction of the manuscript because it is a complete and clear introduction to the topic (it is a good point for someone who knows nothing about this matter). Also, the flow chart and tables are well executed, allowing a good interpretation of the data.

Response: Thank you.

I would like to give you some recommendation to improve your manuscript:

1. Be carefully with the acronyms. You should introduce them in the first mention and keep constant (it can be applied to all the manuscript). Please, check it (especially, in the introduction and discussion sections).

Response: Acronyms are checked and refined.

2. Please, check the English grammar and corrected (only a few words along the manuscript).

Response: Grammar is checked and refined.

3. The information about the ethical approval was duplicated (one in the ethical approval section and the other in the data collection section). Please, check it and eliminate one of them.

Response: It’s been eliminated.

4. I suggest creating a “statistical analysis” section and include both sample size information and statistical test performed in the study. In this way, it looks more organised.

Response: It’s been added.

5. I think that you should provide one or some references regarding your corneal staining grading. Is there any other study that performed in the same way you performed? Or, instead, if you decided to perform according to this method, why? Is it better than others reported in the literature? Please, add some information.

Response: They have been added and color coded in red. 

I have missed more information about the evaluation of the staining (Did you leave the eyes open or close during this time? It is supposed that the patient cannot blink…therefore…how it takes into account? When you perform the measurements in those patients? During those 15 minutes of corneal oxygenation?).

Response: As we have mentioned, all interventions were provided over a 5-day period and the OSD incidence and severity were measured at baseline and post-intervention. Therefore, the eye examinations did not overlap with the interventions. 

6. In methods, it is important to provide the manufactures (small detail) of the product/treatments that you have used. For example, authors have mentioned the use of fluorescein…you should add information about the volume/type of fluorescein (Did you use a strip with saline solution? or instead, a specific volume of fluorescein?). You should think that maybe other researchers want to reproduce your protocol…so, you must provide all the details. Please, applied to all the instruments or devices you used during your research.

Response: They have been added and color coded in red.

7. Regarding the discussion, I think it is correct (also in length) but I think could be improved. I suggest you start talking about your findings, follow by the comparison between treatments (previous studies), follow by the relevance of Polyethylene eye cover and finally your conclusions. I think you should highlight better the findings of your study (it does not seem as much supported by evidence than your introduction).

Response: It has been modified and colored in red.

Additionally, I would like to express some impressions and comments about the manuscript.

1. Despite the great work that you have done…I’m little bit disappointed regarding ocular examination. I think that the evaluation could be more complete than only the corneal damage assessment. Despite keratopathy is the principal ocular complication, both chemosis and microbial keratitis are important in this population.

In fact, this method is particularly useful in patients with chemosis and lagopthalmos (according to the literature, especially including mechanical ventilated patients). Therefore, I think that the assessment of conjunctival integrity (staining and hyperaemia), as well as tear volume, could add valuable information. Previous studies have also included conjunctiva cultures which is a good approach to confirm the microbial presence (very important in this population due to the time that must spent in this condition). I suggest you include a more clinical and diagnostic test in your next study to provide a wider evaluation of the ocular surface.

Response: Yes, you are right. Further studies are required to consider the chemosis, exudate, tear volume, dry eyes, and microbial keratitis as the eye-care intervention outcomes.

2. Regarding the treatment that you choose to compare…I have some doubts. I understand that you include normal saline because it is included in the routine care in Iran and it is your “gold standard”. Despite this, it is well-known (as you mention as well in your introduction and discussion sections) that normal saline is no well supported because of its consequences in the ocular surface. For this reason, it is more than evident that compare this treatment with the others…it will show worst results. In addition to it, it has been proved in the literature (that artificial tears are better in comparison to normal saline and even Polyethylene eye cover versus artificial tears). Therefore, I think the results are quite expected. I think that it would be more interesting to compare two or three treatments that previously have shown a similar behaviour in this population and evaluate which one it is better.

Response: Unfortunately, not only in Iran but in many countries the NS irrigation is common and sometimes is the only routine eye-care. This study reconfirmed the effectiveness of the polyethylene eye-cover and explained how to cover the eyes in detail. More studies are needed for determining the schedule of exchanging eye covers in order to maintain the eyes’ surface oxygenation. 

Reviewer #2: The authors consider an interesting design in this manuscript to assess the efficacy of polyethylene eye cover in preventing occular surface diseases. The statistical study design (2-staged randomization) considered, and the analysis plan, although looking OK, require further clarification, along the lines stated below:

Response: Thank you.

(1) The study is multicenter, however, that structure is not considered in the calculation of the statistical power, or the analysis aspects. To be specific, more argument is necessary on the iid (independent and identically distributed) assumptions of data generated from the 3 ICUs. I mean, can they be considered homogenous? If not, one may need to resort to application of more complex tests, like the clustered McNemar test (see reference a below), or the clustered nonparametric rank sum/Kruskal-Wallis tests (see reference b).

References:

(a) https://www.ncbi.nlm.nih.gov/pubmed/15236431

(b) https://www.tandfonline.com/doi/abs/10.1198/016214504000001583

Response: You are right. The ICU patients are complex and different even in the same ICU, and that is why we considered only one eye of every patient to each study arm. In this way we tried to control the possible differences among participants such as clinical and settings conditions that may influence the eyes’ status.

As shown in table 3, we have compared every single eye with each other, not patients. .

(2) Provide the full name of K-S (I assume Kolmogorov-Smirnov) test when introduced for the first time, in Page 8

Response: replaced.

(3) The sample size calculation uses a proportion test; for more clarity, provide justification of using such a test, including the effect-size considered. The outcome you are considering (such as the OSD severity, etc) are not really proportion responses, right? So, is this calculation appropriate, or you need to use the correct test for calculations?

Response: We set the sample size calculation on the incidence (proportion) of OSD in comatose patients. We also explain the post hoc effect size for the power of test in the text and colored in red, as follow: 

In a post hoc power analysis by G*Power 3.1.9.2 software, when we set a small effect size 0.25, a significance level of α = .05, with total sample size 158 in three groups, the achieved power computed more than 0.80. 

Also, the current version is full of typos, and misconstructs; those need to be corrected. Some are:

. confidential level should be confidence level

. Page 2, Introduction, 3rd sentence: Remove "to"

. Page 5, penultimate paragraph, change "applicable" to "apply".

There are several others, and I leave it upon the authors to rectify.

Response: They are corrected.

 ________________________________________

Attachment Submitted filename: WITHOUT TABLE Response to reviewers - Copy.docx Click here for additional data file.

# Peer review: Decision Letter 1

**DOI:** 10.1371/journal.pone.0248830.r003

**Authors:** Joseph Donlan

3 Nov 2020

PONE-D-19-33881R1

Polyethylene eye cover versus artificial teardrops in the prevention of ocular surface diseases in comatose patients: a prospective multicenter randomized triple-blinded three-arm clinical trial

PLOS ONE

Dear Dr. Falahinia,

Thank you for submitting your manuscript to PLOS ONE - please accept my apologies for the extended delay in getting this decision to you. After careful consideration of your manuscript, and consultation with additional reviewers, we feel that it has merit but still does not fully meet PLOS ONE’s publication criteria as it currently stands. Therefore, we invite you to submit a revised version of the manuscript that addresses the points raised during the review process.

The manuscript has been assessed by the reviewers from the first round, plus two additional external experts, whose comments are appended to this letter. Please review and respond to each of the comments carefully and submit your revised manuscript by Dec 16 2020 11:59PM. If you will need more time than this to complete your revisions, please reply to this message or contact the journal office at plosone@plos.org. When you're ready to submit your revision, log on to https://www.editorialmanager.com/pone/ and select the 'Submissions Needing Revision' folder to locate your manuscript file.

Please include the following items when submitting your revised manuscript:

- A rebuttal letter that responds to each point raised by the academic editor and reviewer(s). You should upload this letter as a separate file labeled 'Response to Reviewers'.
- A marked-up copy of your manuscript that highlights changes made to the original version. You should upload this as a separate file labeled 'Revised Manuscript with Track Changes'.
- An unmarked version of your revised paper without tracked changes. You should upload this as a separate file labeled 'Manuscript'.

If you would like to make changes to your financial disclosure, please include your updated statement in your cover letter. Guidelines for resubmitting your figure files are available below the reviewer comments at the end of this letter.

If applicable, we recommend that you deposit your laboratory protocols in protocols.io to enhance the reproducibility of your results. Protocols.io assigns your protocol its own identifier (DOI) so that it can be cited independently in the future. For instructions see: http://journals.plos.org/plosone/s/submission-guidelines#loc-laboratory-protocols

We look forward to receiving your revised manuscript.

Kind regards,

Dr Joseph Donlan

Staff Editor

PLOS ONE

[Note: HTML markup is below. Please do not edit.]

Reviewers' comments:

Reviewer's Responses to Questions

**Comments to the Author**

1. If the authors have adequately addressed your comments raised in a previous round of review and you feel that this manuscript is now acceptable for publication, you may indicate that here to bypass the “Comments to the Author” section, enter your conflict of interest statement in the “Confidential to Editor” section, and submit your "Accept" recommendation.

Reviewer #1: All comments have been addressed

Reviewer #2: All comments have been addressed

Reviewer #3: All comments have been addressed

Reviewer #4: All comments have been addressed

**********

2. Is the manuscript technically sound, and do the data support the conclusions?

The manuscript must describe a technically sound piece of scientific research with data that supports the conclusions. Experiments must have been conducted rigorously, with appropriate controls, replication, and sample sizes. The conclusions must be drawn appropriately based on the data presented. 

Reviewer #1: Yes

Reviewer #2: (No Response)

Reviewer #3: Partly

Reviewer #4: Partly

**********

3. Has the statistical analysis been performed appropriately and rigorously? 

Reviewer #1: Yes

Reviewer #2: (No Response)

Reviewer #3: Yes

Reviewer #4: Yes

**********

4. Have the authors made all data underlying the findings in their manuscript fully available?

The PLOS Data policy requires authors to make all data underlying the findings described in their manuscript fully available without restriction, with rare exception (please refer to the Data Availability Statement in the manuscript PDF file). The data should be provided as part of the manuscript or its supporting information, or deposited to a public repository. For example, in addition to summary statistics, the data points behind means, medians and variance measures should be available. If there are restrictions on publicly sharing data—e.g. participant privacy or use of data from a third party—those must be specified.

Reviewer #1: Yes

Reviewer #2: (No Response)

Reviewer #3: Yes

Reviewer #4: Yes

**********

5. Is the manuscript presented in an intelligible fashion and written in standard English?

PLOS ONE does not copyedit accepted manuscripts, so the language in submitted articles must be clear, correct, and unambiguous. Any typographical or grammatical errors should be corrected at revision, so please note any specific errors here.

Reviewer #1: Yes

Reviewer #2: (No Response)

Reviewer #3: Yes

Reviewer #4: Yes

**********

6. Review Comments to the Author

Please use the space provided to explain your answers to the questions above. You may also include additional comments for the author, including concerns about dual publication, research ethics, or publication ethics. (Please upload your review as an attachment if it exceeds 20,000 characters)

Reviewer #1: Thank you to authors to take into account all the suggestions made.

Despite most of the comments have been addressed, several grammar mistakes, repeated words or wrong spelling still remain in the manuscript.Please check it again.

Here I detail some of them:

Line 9 (INTRODUCTION): Conjunctival infections to. serious everlasting...

Line 22 (INTRODUCTION): Applying polyethylene eye covers has (BEEN) suggested as the most...

Line 26 (INTRODUCTION): So that, the standard care has been only COMPRISED COMPROMISED a daily washing...

Fig 1: Fallow-up (Follow-up)

Discussion: Our findings are also...by Sahn and Min in showing...as found by Shan and Min (redundant due to repetition)

and more...Please, take a look at these type of errors.

Reviewer #2: (No Response)

Reviewer #3: Polyethylene eye-cover versus artificial teardrops in the prevention of ocular surface diseases in comatose patients: a prospective multicenter randomized triple-blinded three-arm clinical trial

The authors have compared using normal saline eye drop versus lubricant eye drops versus polyethylene eye covers for study of ocular surface disease in comatose patients. Though it is an important topic to be addressed, there are some basic issues with the manuscript. The results are expected, and add limited knowledge to existing literature.

Major comments:

1. Page 4, line 5: “So, that,,,” This line appears to be misquoted. The standard care for authors of reference 20 was that. It cannot be considered as the universal standard of care. Standards should be quoted based on guidelines – for example, The Royal college guidelines: https://www.rcophth.ac.uk/wp-content/uploads/2017/11/Intensive-Care-Unit.pdf. In fact, these guidelines are against the usage of NS, and suggest warm water bath.

2. Page 4, paragraph 2, line 1: “In Iran…” This line and the authors’ reply to reviewer 1 states that NS washing is routine eye care in ICU. Below is a paper from Iran which also proposes against the use of NS.

Impact of Normal Saline on the Incidence of Exposure Keratopathy in Patients Hospitalized in Intensive Care Units. Iran J Nurs Midwifery Res. 2018 Jan-Feb; 23(1): 57–60. doi: 10.4103/ijnmr.IJNMR_187_16

It is ironic why it is still continued as a routine care in Iran, despite being proven adverse. The authors also acknowledge this in their critical appraisal, yet have continued to do so, which is disappointing.

3. Was it the same blinded examiner who studied the patients in all the 3 ICUs?

4. Including all comatose patients in this study could have led to bias. For example, a cornea exposed eye being administered just 2 drops of NS Q6H, is likely to develop OSD while only conjunctiva exposed eye or a closed eye may not.

As per standard guidelines (Ref: https://www.rcophth.ac.uk/wp-content/uploads/2017/11/Intensive-Care-Unit.pdf), eyes which have grade 0 closure need no action at all, and eyes with only conjunctival exposure (grade-1) will have unaffected corneas even otherwise. Since the authors are only looking for corneal manifestations, ideally only grade-2 exposed eyes should have been included.

5. It is said that all eyes had a healthy cornea (confirmed by ophthalmologist) at the start of the study. After institution of therapy, when were they re-examined – at the end of 5 day period? Was the examination time kept constant for all eyes, like for example, 1 hour after last therapy?

6. Elaborating the results on how many eyes had +1 grading due to the mentioned criteria, and their breakdown could be informative.

7. A photograph of the polyethylene film applied can make the manuscript more appealing.

8. As per reviewer 1’s comments and my concern, it is important to add on to limitations about missing on the other forms of OSD in unconscious patients such as epithelial defects, infections, tear studies and conjunctival manifestations.

9. References 17 & 19 are the same. Please remove one.

10. Were they tear drops or ointments – please provide the type and concentration of the lubricant along with manufacturer details.

11. Supplementary detailed results, table 7, 8: Please either give row % or column %. The combination of both appears confusing.

12. Supplementary detailed results, table 9: The data between table 9 and table 7,8 does not tally for artificial tear drops and polyethylene cover. For example, table 8 shows a total of 8 OSD eyes, but table 9 shows only 7 OSD cases.

13. For table 3 and supplementary table 9 and 15: Please give the individual p values between the groups.

14. In my opinion, the discussion needs to stress against the use of NS drops/irrigation so that the practice in Iran could change.

Minor comments:

Introduction line 4: but ‘receive’ minimal

Introduction line 7: Do the authors mean ‘altered’ tear components concentration?

Page 3, paragraph 2, line 4: I think washing eyes with NS should come last, as it is proven to show adverse effects.

Figure 1: correct ‘follow-up’

Figure 1: ‘lost to follow-up due to meet exclusion criteria’ – rephrase/explain

Table 1: Does Internal patients mean internal medicine department patients? Please clarify internal patients

Page 13, line 4 appears out of context. May be more suitable in the subsequent paragraph.

Page 13, line 14: Pls mention what was the other intervention

Page 13, line 18: Recheck the sentence

Page 13, last paragraph first line: This line is misleading. Reference 4 clearly states against cotton wool balls and NS irrigation in its ‘DO-NOTs’ section.

Page 14, line 7: ‘pre-disposable’ polyethylene eye cover – meaning?

Supporting information file: ‘original’ results

Reviewer #4: The article in question addresses a topic of extreme relevance for eye health in critically ill patients, as eye care in intensive care units is often postponed due to little knowledge by nurses and the multidisciplinary team about the anatomy and physiology of the eye, evaluation and the care that can be implemented to avoid possible damage.

However, in order to confer greater scientific criticality and greater intelligibility of the text of the article, I describe some considerations below:

1.Title: Clear, attractive and relevant to the text.

2.Abstract: Well structured and coherent.

3.Keywords: I suggest modifying “Infectious Diseases” by “Eye Diseases”, as diseases of the ocular surface are not limited to infectious diseases.

4. Introduction

Although well described, targeted and synthetic, I missed a definition of ocular surface diseases in the introduction and possible diagnostic methods. In addition, it needs to describe more clearly what the current study shows different/new when compared to other clinical trials that also compared polyethylene covers with artificial tears.

5. Materials and methods

Describe more clearly how ocular surface disease was established/diagnosed and what degrees of fluorescein testing were considered to classify OSD?

Justify the reason for having followed up for a period of five days. In addition, it is not clear whether all patients were followed up entirely during the five days or whether they were followed up until the appearance of changes in the fluorescein test indicating the presence of the outcome (OSD).

It was not clear at what time the cornea was evaluated with fluorescein staining. Was it used daily? At the same times for all patients? What procedures were performed before the application of fluorescein that allowed the blinding of the ophthalmologist?

In data collection, when the researchers describe the data on the demographic and clinical characteristics of the patients (Age, sex and type of injury), in fact, what appears in the results is not the type of injury, but the reason for ICU admission. Then, I suggest adjusting this information.

I suggest describing manufacturers/specific brands of interventions/treatments (Polyethylene cover, artificial teardrops and normal saline), as well as briefly describing the presentation characteristics of each one.

6. Results

According to CONSORT, the flow diagram must be presented in the results.

What does “Internal patients” mean for reasons of hospitalization?

The information " The OSDs were observed in 66 (41.77%) out of 158 eyes examined" appears only in the abstract of the work. Therefore, it must also be included in the search results.

In the results, the authors demonstrated the presence or not of statistical association between comparisons of interventions. However, they did not indicate/describe the magnitude of the association, using, for example, the Relative Risk with their respective confidence intervals.

7. Discussion

It provides a logical and appropriate interpretation of results in an expanded scientific context and manages to engage in a targeted dialogue with literature and other studies.

8. Conclusion

Precise, clear and consistent with the purpose of the study.

9. References

Relevant and appropriate to the text and theme.

**********

7. PLOS authors have the option to publish the peer review history of their article (what does this mean?). If published, this will include your full peer review and any attached files.

If you choose “no”, your identity will remain anonymous but your review may still be made public.

**Do you want your identity to be public for this peer review?** For information about this choice, including consent withdrawal, please see our Privacy Policy.

Reviewer #1: No

Reviewer #2: No

Reviewer #3: No

Reviewer #4: No

[NOTE: If reviewer comments were submitted as an attachment file, they will be attached to this email and accessible via the submission site. Please log into your account, locate the manuscript record, and check for the action link "View Attachments". If this link does not appear, there are no attachment files.]

While revising your submission, please upload your figure files to the Preflight Analysis and Conversion Engine (PACE) digital diagnostic tool, https://pacev2.apexcovantage.com/. PACE helps ensure that figures meet PLOS requirements. To use PACE, you must first register as a user. Registration is free. Then, login and navigate to the UPLOAD tab, where you will find detailed instructions on how to use the tool. If you encounter any issues or have any questions when using PACE, please email PLOS at figures@plos.org. Please note that Supporting Information files do not need this step.

# Author comment: Author response to Decision Letter 1

**DOI:** 10.1371/journal.pone.0248830.r004

25 Nov 2020

- Reviewer #1: 

- Thank you to authors to take into account all the suggestions made.

Response: Thank you too.

- Despite most of the comments have been addressed, several grammar mistakes, repeated words or wrong spelling still remain in the manuscript. 

Response: Corrected.

- Reviewer #2: Nothing

Response: -

- Reviewer #3:

- The authors have compared using normal saline eye drop versus lubricant eye drops versus polyethylene eye covers for study of ocular surface disease in comatose patients. Though it is an important topic to be addressed, there are some basic issues with the manuscript. The results are expected, and add limited knowledge to existing literature. 

Response: As far as the authors know, this is the first prospective multicenter randomized triple-blinded three-arm clinical trial in which each patient was a member of the control and experimental group simultaneously. Furthermore, we considered changing the eye-covers every 12 hours with a 15 minutes lag for corneal oxygenation, not like the other studies which had a 24-hour schedule without considering the natural cornea oxygenation. We studied both the incidence and severity of OSD in ICU patients. However, there are many spaces for such a study, and we think that explaining the methodology in detail can help and facilitate future studies for other eye care methods in ICUs.

- 1. Page 4, line 5: “So, that,,,” This line appears to be misquoted. The standard care for authors of reference 20 was that. It cannot be considered as the universal standard of care. Standards should be quoted based on guidelines – for example, The Royal college guidelines: https://www.rcophth.ac.uk/wp-content/uploads/2017/11/Intensive-Care-Unit.pdf. In fact, these guidelines are against the usage of NS, and suggest warm water bath. 

Response: Corrected.

- 2. Page 4, paragraph 2, line 1: “In Iran…” This line and the authors’ reply to reviewer 1 states that NS washing is routine eye care in ICU. Below is a paper from Iran which also proposes against the use of NS.

Impact of Normal Saline on the Incidence of Exposure Keratopathy in Patients Hospitalized in Intensive Care Units. Iran J Nurs Midwifery Res. 2018 Jan-Feb; 23(1): 57–60. doi: 10.4103/ijnmr.IJNMR_187_16

It is ironic why it is still continued as a routine care in Iran, despite being proven adverse. The authors also acknowledge this in their critical appraisal, yet have continued to do so, which is disappointing. 

Response: NS is not recommended anymore, yet unfortunately, it is routine care in the ICUs in Iran and many in developing countries. As we mentioned in the text, we added NS drops, teardrops, and eye covering to the routine care (irrigation the eyes with NS). So, the first step in our three ways of interventions was very gentle lids and skin washing by NS. Your comments will be a good idea for future clinical trials about using sterile water instead of NS to clean the lids and crusts. 

- 3. Was it the same blinded examiner who studied the patients in all the 3 ICUs? 

Response: Yes. It was written as below in text: A single observer who was blinded as to the patient assignments evaluated the patients’ eyes based on the Corneal Fluorescein Staining Pattern. 

- 4. Including all comatose patients in this study could have led to bias. For example, a cornea exposed eye being administered just 2 drops of NS Q6H, is likely to develop OSD while only conjunctiva exposed eye or a closed eye may not.

As per standard guidelines (Ref: https://www.rcophth.ac.uk/wp-content/uploads/2017/11/Intensive-Care-Unit.pdf), eyes which have grade 0 closure need no action at all, and eyes with only conjunctival exposure (grade-1) will have unaffected corneas even otherwise. Since the authors are only looking for corneal manifestations, ideally only grade-2 exposed eyes should have been included. 

Response: As we mentioned, an ophthalmologist confirmed whether the patients’ eyes had the study inclusion criteria or not. Comparing was done between two eyes of the same patient who was simultaneously under 2 methods, so, the exposure of both eyes was the same. 

- 5. It is said that all eyes had a healthy cornea (confirmed by ophthalmologist) at the start of the study. After institution of therapy, when were they re-examined – at the end of 5 day period? Was the examination time kept constant for all eyes, like for example, 1 hour after last therapy? 

Response: Explained.

- 6. Elaborating the results on how many eyes had +1 grading due to the mentioned criteria, and their breakdown could be informative. 

Response: Please find Table 3 where we wrote the number of the eyes with different severity grades of OSD criteria in each group separately.

- 7. A photograph of the polyethylene film applied can make the manuscript more appealing. 

Response: The procedure of eye covering was according to what was advised in the references bellow: 

Sharjeel M, Malik IQ, Iqbal CJ, Ali F. Prevention of exposure keratopathy with sahaf wet chamber. Pakistan Journal of Ophthalmology. 2015;31(3):131-6.

Al-Ribh SA, Baker RH, Gupta RK, Al Dossary TS. Polyethylene cover versus viscotears gel for the prevention of corneal abrasions in critically ill patients: a comparative study. Journal of King Abdulaziz University-Medical Sciences. 2012;19(1 Sup):59-72. doi: https://doi.org/10.4197/Med.19-1S.4.

- 8. As per reviewer 1’s comments and my concern, it is important to add on to limitations about missing on the other forms of OSD in unconscious patients such as epithelial defects, infections, tear studies and conjunctival manifestations. 

Response: Added.

- 9. References 17 & 19 are the same. Please remove one. 

Response: Corrected.

- 10. Were they tear drops or ointments – please provide the type and concentration of the lubricant along with manufacturer details. 

Response: Added.

- 11. Supplementary detailed results, table 7, 8: Please either give row % or column %. The combination of both appears confusing. .

Response: Corrected.

- 12. Supplementary detailed results, table 9: The data between table 9 and table 7,8 does not tally for artificial tear drops and polyethylene cover. For example, table 8 shows a total of 8 OSD eyes, but table 9 shows only 7 OSD cases. 

Response: Corrected.

- 13. For table 3 and supplementary table 9 and 15: Please give the individual p values between the groups. 

Response: Tables of comparing of the incidence and severity between two groups are added in the supplementary. 

- 14. In my opinion, the discussion needs to stress against the use of NS drops/irrigation so that the practice in Iran could change.

Response: Added.

- Minor comments: Introduction line 4: but ‘receive’ minimal.

Response: Corrected.

- Introduction line 7: Do the authors mean ‘altered’ tear components concentration? 

Response: Corrected.

- Page 3, paragraph 2, line 4: I think washing eyes with NS should come last, as it is proven to show adverse effects.

NS irrigation was introduced by many references we used for that paragraph (References 3, 4, 8, 10, 12-17). So, may be better to come first. 

- Figure 1: correct ‘follow-up’ 

Response: Corrected.

- Figure 1: ‘lost to follow-up due to meet exclusion criteria’ – rephrase/explain 

Response: Explained.

- Table 1: Does Internal patients mean internal medicine department patients? Please clarify internal patients.

Response: Explained.

- Page 13, line 4 appears out of context. May be more suitable in the subsequent paragraph. 

Response: Corrected.

- Page 13, line 14: Pls mention what was the other intervention 

Response: Explained.

- Page 13, line 18: Recheck the sentence 

Response: Corrected.

- Page 13, last paragraph first line: This line is misleading. Reference 4 clearly states against cotton wool balls and NS irrigation in its ‘DO-NOTs’ section. 

Response: Corrected.

- Page 14, line 7: ‘pre-disposable’ polyethylene eye cover – meaning? 

Response: We cut pieces of 10*10 polyethylene film (clear plastic film) by scissors for every patient and fixed them over the patients’ eyes with adhesive tapes. If these plastic pieces with the edge adhesive ability were ready, the time and using them would have been lesser and easier.

- Supporting information file: ‘original’ results 

Response: Added.

Reviewer #4: 

- However, in order to confer greater scientific criticality and greater intelligibility of the text of the article, I describe some considerations below:

- 1.Title: Clear, attractive and relevant to the text. 

Response: Thank you.

- 2.Abstract: Well structured and coherent. 

Response: Thank you.

- 3.Keywords: I suggest modifying “Infectious Diseases” by “Eye Diseases”, as diseases of the ocular surface are not limited to infectious diseases. 

Response: Modified.

- 4. Introduction

- Although well described, targeted and synthetic, I missed a definition of ocular surface diseases in the introduction and possible diagnostic methods.

Response: Added.

- In addition, it needs to describe more clearly what the current study shows different/new when compared to other clinical trials that also compared polyethylene covers with artificial tears. 

As far as the authors know, 

1- This is the first prospective multicenter randomized triple-blinded three-arm clinical trial in this regards.

2- During the study, we considered changing the eye-covers every 12 hours with a 15 minutes lag for corneal oxygenation. Other studies had a 24-hour schedule without considering the cornea oxygenation.

3- In our study, each patient was a member of the control and/or experimental group simultaneously. 

4- We studied both the incidence and severity of OSD in ICU patients in which the results showed both incidence and severity of OSD depended on the eye care method. 

However, there are many spaces for such a study, and we think that explaining the methodology in detail can help and facilitate future studies for other eye care methods in ICUs.

- 5. Materials and methods

- Describe more clearly how ocular surface disease was established/diagnosed and what degrees of fluorescein testing were considered to classify OSD?

Response: Explained.

- Justify the reason for having followed up for a period of five days. In addition, it is not clear whether all patients were followed up entirely during the five days or whether they were followed up until the appearance of changes in the fluorescein test indicating the presence of the outcome (OSD).

Response: Added. 

- It was not clear at what time the cornea was evaluated with fluorescein staining. Was it used daily? At the same times for all patients? What procedures were performed before the application of fluorescein that allowed the blinding of the ophthalmologist?

Response: Explained.

- In data collection, when the researchers describe the data on the demographic and clinical characteristics of the patients (Age, sex and type of injury), in fact, what appears in the results is not the type of injury, but the reason for ICU admission. Then, I suggest adjusting this information.

Response: Corrected.

- I suggest describing manufacturers/ specific brands of interventions/ treatments (Polyethylene cover, artificial teardrops and normal saline), as well as briefly describing the presentation characteristics of each one. 

Response: In our home university, we are inhibited to name the brand of materials because of advertisement and potential conflict of interest. However, more details of these materials are added.

- 6. Results

- According to CONSORT, the flow diagram must be presented in the results. 

Response: Replaced.

- What does “Internal patients” mean for reasons of hospitalization? 

Response: Explained.

- The information " The OSDs were observed in 66 (41.77%) out of 158 eyes examined" appears only in the abstract of the work. Therefore, it must also be included in the search results. 

Response: Added.

- In the results, the authors demonstrated the presence or not of statistical association between comparisons of interventions. However, they did not indicate/describe the magnitude of the association, using, for example, the Relative Risk with their respective confidence intervals. 

Response: Tables of comparing of the incidence and severity between two groups are added in the supplementary. 

- 7. Discussion

- It provides a logical and appropriate interpretation of results in an expanded scientific context and manages to engage in a targeted dialogue with literature and other studies.

Response: Thank you.

- 8. Conclusion

- Precise, clear and consistent with the purpose of the study.

Response: Thank you.

- 9. References

- Relevant and appropriate to the text and theme. 

Response: Thank you.

Attachment Submitted filename: Second-Responses to the reviewers .docx Click here for additional data file.

# Peer review: Decision Letter 2

**DOI:** 10.1371/journal.pone.0248830.r005

**Authors:** Natasha McDonald

15 Jan 2021

PONE-D-19-33881R2

Polyethylene eye cover versus artificial teardrops in the prevention of ocular surface diseases in comatose patients: a prospective multicenter randomized triple-blinded three-arm clinical trial

PLOS ONE

Dear Dr. Falahinia,

Thank you for submitting your manuscript to PLOS ONE. After careful consideration, we feel that it has merit but does not fully meet PLOS ONE’s publication criteria as it currently stands. Therefore, we invite you to submit a revised version of the manuscript that addresses the points raised during the review process.

A few of the reviewers' concerns remain unresolved and should be thoroughly addressed. In particular, Reviewer 3 felt that their comment about the inclusion of comatose patients needs to be resolved. They also felt that their suggested changes to the supplemental tables should be addressed. Furthermore, we feel that your manuscript requires editing for English grammar and language. The reviewers' comments can be viewed in full, below.

Please submit your revised manuscript by Feb 28 2021 11:59PM. If you will need more time than this to complete your revisions, please reply to this message or contact the journal office at plosone@plos.org. When you're ready to submit your revision, log on to https://www.editorialmanager.com/pone/ and select the 'Submissions Needing Revision' folder to locate your manuscript file.

Please include the following items when submitting your revised manuscript:

- A rebuttal letter that responds to each point raised by the academic editor and reviewer(s). You should upload this letter as a separate file labeled 'Response to Reviewers'.
- A marked-up copy of your manuscript that highlights changes made to the original version. You should upload this as a separate file labeled 'Revised Manuscript with Track Changes'.
- An unmarked version of your revised paper without tracked changes. You should upload this as a separate file labeled 'Manuscript'.

If you would like to make changes to your financial disclosure, please include your updated statement in your cover letter. Guidelines for resubmitting your figure files are available below the reviewer comments at the end of this letter.

If applicable, we recommend that you deposit your laboratory protocols in protocols.io to enhance the reproducibility of your results. Protocols.io assigns your protocol its own identifier (DOI) so that it can be cited independently in the future. For instructions see: http://journals.plos.org/plosone/s/submission-guidelines#loc-laboratory-protocols

We look forward to receiving your revised manuscript.

Kind regards,

Natasha McDonald, PhD

Associate Editor

PLOS ONE

[Note: HTML markup is below. Please do not edit.]

Reviewers' comments:

Reviewer's Responses to Questions

**Comments to the Author**

1. If the authors have adequately addressed your comments raised in a previous round of review and you feel that this manuscript is now acceptable for publication, you may indicate that here to bypass the “Comments to the Author” section, enter your conflict of interest statement in the “Confidential to Editor” section, and submit your "Accept" recommendation.

Reviewer #1: All comments have been addressed

Reviewer #2: All comments have been addressed

Reviewer #3: (No Response)

**********

2. Is the manuscript technically sound, and do the data support the conclusions?

The manuscript must describe a technically sound piece of scientific research with data that supports the conclusions. Experiments must have been conducted rigorously, with appropriate controls, replication, and sample sizes. The conclusions must be drawn appropriately based on the data presented. 

Reviewer #1: Yes

Reviewer #2: Yes

Reviewer #3: Yes

**********

3. Has the statistical analysis been performed appropriately and rigorously? 

Reviewer #1: Yes

Reviewer #2: (No Response)

Reviewer #3: Yes

**********

4. Have the authors made all data underlying the findings in their manuscript fully available?

The PLOS Data policy requires authors to make all data underlying the findings described in their manuscript fully available without restriction, with rare exception (please refer to the Data Availability Statement in the manuscript PDF file). The data should be provided as part of the manuscript or its supporting information, or deposited to a public repository. For example, in addition to summary statistics, the data points behind means, medians and variance measures should be available. If there are restrictions on publicly sharing data—e.g. participant privacy or use of data from a third party—those must be specified.

Reviewer #1: Yes

Reviewer #2: Yes

Reviewer #3: Yes

**********

5. Is the manuscript presented in an intelligible fashion and written in standard English?

PLOS ONE does not copyedit accepted manuscripts, so the language in submitted articles must be clear, correct, and unambiguous. Any typographical or grammatical errors should be corrected at revision, so please note any specific errors here.

Reviewer #1: Yes

Reviewer #2: Yes

Reviewer #3: Yes

**********

6. Review Comments to the Author

Please use the space provided to explain your answers to the questions above. You may also include additional comments for the author, including concerns about dual publication, research ethics, or publication ethics. (Please upload your review as an attachment if it exceeds 20,000 characters)

Reviewer #1: The authors have addressed all of my recommendations. Together with the rest of the reviewers' recommendations, the manuscript has been improved. As a final comment, I suggest the authors check the acronyms again. Be sure that all of them were defined previously.

Reviewer #2: (No Response)

Reviewer #3: • An English revision is required.

• I doubt if my previous comment #4 was misunderstood: “Including all comatose patients in this study could have led to bias. For example, a cornea exposed eye being administered just 2 drops of NS Q6H, is likely to develop OSD while only conjunctiva exposed eye or a closed eye may not. As per standard guidelines (Ref: https://www.rcophth.ac.uk/wpcontent/uploads/2017/11/Intensive-Care-Unit.pdf), eyes which have grade 0 closure need no action at all, and eyes with only conjunctival exposure (grade-1) will have unaffected corneas even otherwise. Since the authors are only looking for corneal manifestations, ideally only grade-2 exposed eyes should have been included”. I meant that including only cornea exposed eyes would have given more specific results, and did not mean the inter-eye symmetry. Or, the exposure should have been graded 0-2, and each grade analyzed individually. The inclusion criteria the authors have stated appear to be primarily related to their inability to blink and a normal cornea at the start.

However, this is a bias related to the core of the study, and I would suggest the authors to add this point under the limitations.

• Previous comment #6 was questioned about “A score of severity is added when the PEE was seen in the central 4mm diameter portion of the cornea, one or more filaments occurred anywhere on the cornea, or one or more patches of confluent staining, including linear stains, are found anywhere on the cornea”

• Previous last minor comment was to replace ‘origin’ results by ‘original’ results at the legend for supporting file. I’m sorry that it was misunderstood. The present supplementary tables are too numerous and confusing, please keep the previous tables itself, but correct the numerical errors as explained previously.

**********

7. PLOS authors have the option to publish the peer review history of their article (what does this mean?). If published, this will include your full peer review and any attached files.

If you choose “no”, your identity will remain anonymous but your review may still be made public.

**Do you want your identity to be public for this peer review?** For information about this choice, including consent withdrawal, please see our Privacy Policy.

Reviewer #1: No

Reviewer #2: No

Reviewer #3: No

[NOTE: If reviewer comments were submitted as an attachment file, they will be attached to this email and accessible via the submission site. Please log into your account, locate the manuscript record, and check for the action link "View Attachments". If this link does not appear, there are no attachment files.]

While revising your submission, please upload your figure files to the Preflight Analysis and Conversion Engine (PACE) digital diagnostic tool, https://pacev2.apexcovantage.com/. PACE helps ensure that figures meet PLOS requirements. To use PACE, you must first register as a user. Registration is free. Then, login and navigate to the UPLOAD tab, where you will find detailed instructions on how to use the tool. If you encounter any issues or have any questions when using PACE, please email PLOS at figures@plos.org. Please note that Supporting Information files do not need this step.

# Author comment: Author response to Decision Letter 2

**DOI:** 10.1371/journal.pone.0248830.r006

30 Jan 2021

Revised portion are highlighted in the manuscript. The main corrections in the paper and the responds to the Editor’s and reviewer’s comments are as following: 

6. Review Comments to the Author

Reviewer #1: The authors have addressed all of my recommendations. Together with the rest of the reviewers' recommendations, the manuscript has been improved. As a final comment, I suggest the authors check the acronyms again. Be sure that all of them were defined previously.

Response: Thank you very much. The acronyms are checked and refined.

Reviewer #2: (No Response)

Response: -

Reviewer #3: • An English revision is required.

Response: The English editing is checked and refined.

• I doubt if my previous comment #4 was misunderstood: “Including all comatose patients in this study could have led to bias. For example, a cornea exposed eye being administered just 2 drops of NS Q6H, is likely to develop OSD while only conjunctiva exposed eye or a closed eye may not. As per standard guidelines (Ref: https://www.rcophth.ac.uk/wpcontent/uploads/2017/11/Intensive-Care-Unit.pdf), eyes which have grade 0 closure need no action at all, and eyes with only conjunctival exposure (grade-1) will have unaffected corneas even otherwise. Since the authors are only looking for corneal manifestations, ideally only grade-2 exposed eyes should have been included”. I meant that including only cornea exposed eyes would have given more specific results, and did not mean the inter-eye symmetry. Or, the exposure should have been graded 0-2, and each grade analyzed individually. The inclusion criteria the authors have stated appear to be primarily related to their inability to blink and a normal cornea at the start.

However, this is a bias related to the core of the study, and I would suggest the authors to add this point under the limitations.

Response: Although our eligibility criteria were included incomplete eyelids closure too, the grades of eye closure were not our main criterion. So, this point is added to the limitations.

• Previous comment #6 was questioned about “A score of severity is added when the PEE was seen in the central 4mm diameter portion of the cornea, one or more filaments occurred anywhere on the cornea, or one or more patches of confluent staining, including linear stains, are found anywhere on the cornea”

Response: More detail is explained.

• Previous last minor comment was to replace ‘origin’ results by ‘original’ results at the legend for supporting file. I’m sorry that it was misunderstood. The present supplementary tables are too numerous and confusing, please keep the previous tables itself, but correct the numerical errors as explained previously.

Response: Replaced.

*****************

Best regards,

Dr. Mahnaz Khatiban

Attachment Submitted filename: 4th Responses to the reviewers .docx Click here for additional data file.

# Peer review: Decision Letter 3

**DOI:** 10.1371/journal.pone.0248830.r007

**Authors:** Catherine E Oldenburg

8 Mar 2021

Polyethylene eye cover versus artificial teardrops in the prevention of ocular surface diseases in comatose patients: a prospective multicenter randomized triple-blinded three-arm clinical trial

PONE-D-19-33881R3

Dear Dr. Falahinia,

We’re pleased to inform you that your manuscript has been judged scientifically suitable for publication and will be formally accepted for publication once it meets all outstanding technical requirements.

Within one week, you’ll receive an e-mail detailing the required amendments. When these have been addressed, you’ll receive a formal acceptance letter and your manuscript will be scheduled for publication.

An invoice for payment will follow shortly after the formal acceptance. To ensure an efficient process, please log into Editorial Manager at http://www.editorialmanager.com/pone/, click the 'Update My Information' link at the top of the page, and double check that your user information is up-to-date. If you have any billing related questions, please contact our Author Billing department directly at authorbilling@plos.org.

If your institution or institutions have a press office, please notify them about your upcoming paper to help maximize its impact. If they’ll be preparing press materials, please inform our press team as soon as possible -- no later than 48 hours after receiving the formal acceptance. Your manuscript will remain under strict press embargo until 2 pm Eastern Time on the date of publication. For more information, please contact onepress@plos.org.

Kind regards,

Catherine E Oldenburg

Academic Editor

PLOS ONE

Additional Editor Comments (optional):

Reviewers' comments:

Reviewer's Responses to Questions

**Comments to the Author**

1. If the authors have adequately addressed your comments raised in a previous round of review and you feel that this manuscript is now acceptable for publication, you may indicate that here to bypass the “Comments to the Author” section, enter your conflict of interest statement in the “Confidential to Editor” section, and submit your "Accept" recommendation.

Reviewer #2: All comments have been addressed

Reviewer #3: All comments have been addressed

**********

2. Is the manuscript technically sound, and do the data support the conclusions?

The manuscript must describe a technically sound piece of scientific research with data that supports the conclusions. Experiments must have been conducted rigorously, with appropriate controls, replication, and sample sizes. The conclusions must be drawn appropriately based on the data presented. 

Reviewer #2: (No Response)

Reviewer #3: Yes

**********

3. Has the statistical analysis been performed appropriately and rigorously? 

Reviewer #2: (No Response)

Reviewer #3: Yes

**********

4. Have the authors made all data underlying the findings in their manuscript fully available?

The PLOS Data policy requires authors to make all data underlying the findings described in their manuscript fully available without restriction, with rare exception (please refer to the Data Availability Statement in the manuscript PDF file). The data should be provided as part of the manuscript or its supporting information, or deposited to a public repository. For example, in addition to summary statistics, the data points behind means, medians and variance measures should be available. If there are restrictions on publicly sharing data—e.g. participant privacy or use of data from a third party—those must be specified.

Reviewer #2: (No Response)

Reviewer #3: Yes

**********

5. Is the manuscript presented in an intelligible fashion and written in standard English?

PLOS ONE does not copyedit accepted manuscripts, so the language in submitted articles must be clear, correct, and unambiguous. Any typographical or grammatical errors should be corrected at revision, so please note any specific errors here.

Reviewer #2: (No Response)

Reviewer #3: (No Response)

**********

6. Review Comments to the Author

Please use the space provided to explain your answers to the questions above. You may also include additional comments for the author, including concerns about dual publication, research ethics, or publication ethics. (Please upload your review as an attachment if it exceeds 20,000 characters)

Reviewer #2: (No Response)

Reviewer #3: (No Response)

**********

7. PLOS authors have the option to publish the peer review history of their article (what does this mean?). If published, this will include your full peer review and any attached files.

If you choose “no”, your identity will remain anonymous but your review may still be made public.

**Do you want your identity to be public for this peer review?** For information about this choice, including consent withdrawal, please see our Privacy Policy.

Reviewer #2: No

Reviewer #3: No

# Editor report: Acceptance letter

**DOI:** 10.1371/journal.pone.0248830.r008

**Authors:** Catherine E Oldenburg

22 Mar 2021

PONE-D-19-33881R3 

Polyethylene eye-cover versus artificial teardrops in the prevention of ocular surface diseases in comatose patients: a prospective multicenter randomized triple-blinded three-arm clinical trial 

Dear Dr. Falahinia:

I'm pleased to inform you that your manuscript has been deemed suitable for publication in PLOS ONE. Congratulations! Your manuscript is now with our production department. 

If your institution or institutions have a press office, please let them know about your upcoming paper now to help maximize its impact. If they'll be preparing press materials, please inform our press team within the next 48 hours. Your manuscript will remain under strict press embargo until 2 pm Eastern Time on the date of publication. For more information please contact onepress@plos.org.

If we can help with anything else, please email us at plosone@plos.org. 

Thank you for submitting your work to PLOS ONE and supporting open access. 

Kind regards, 

PLOS ONE Editorial Office Staff

on behalf of

Dr. Catherine E Oldenburg 

Academic Editor

PLOS ONE
